# Adding More Shape to Nanoscale Reference Materials—LiYF_4_:Yb,Tm Bipyramids as Standards for Sizing Methods and Particle Number Concentration

**DOI:** 10.1021/acs.analchem.4c03641

**Published:** 2024-11-13

**Authors:** Jérôme Deumer, Elina Andresen, Christian Gollwitzer, Robin Schürmann, Ute Resch-Genger

**Affiliations:** † Physikalisch-Technische Bundesanstalt, Abbestraße 2-12, 10587 Berlin, Germany; ‡ Division Biophotonics, Bundesanstalt für Materialforschung und -prüfung, Richard-Willstaetter-Straße 11, 12489 Berlin, Germany

## Abstract

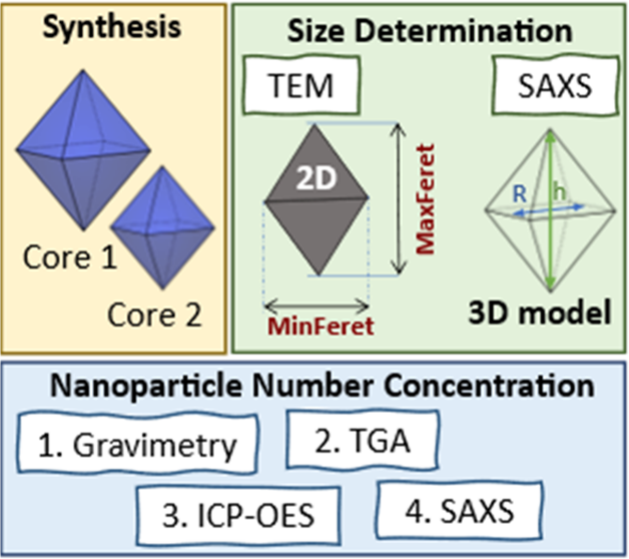

The increasing industrial use of nanomaterials calls for the reliable characterization of their physicochemical key properties like size, size distribution, shape, and surface chemistry, and test and reference materials (RMs) with sizes and shapes, closely matching real-world nonspheric nano-objects. An efficient strategy to minimize efforts in producing nanoscale RMs (nanoRMs) for establishing, validating, and standardizing methods for characterizing nanomaterials are multimethod nanoRMs. Ideal candidates are lanthanide-based, multicolor luminescent, and chemically inert nanoparticles (NPs) like upconversion nanoparticles (UCNPs), which can be prepared in different sizes, shapes, and chemical composition with various surface coatings. This makes UCNPs interesting candidates as standards not only for sizing methods, but also for element-analytical methods like laser ablation-inductively coupled plasma mass spectrometry (LA-ICP-MS), quantitative bioimaging methods like X-ray fluorescence computed tomography (XFCT), and luminescence methods and correlative measurements. Here, we explore the potential of two monodisperse LiYF_4_:Yb,Tm bipyramids with peak-to-peak distances of (43 ± 2) nm and (29 ± 2) nm as size standards for small-angle X-ray scattering (SAXS) and tools for establishing and validating the sophisticated simulations required for the analysis of SAXS data derived from dispersions of nonspheric nano-objects. These SAXS studies are supplemented by two-dimensional (2D)-transmission electron microscopy measurements of the UCNP bipyramids. Additionally, the particle number concentration of cyclohexane dispersions of these UCNP bipyramids is determined by absolute SAXS measurements, complemented by gravimetry, thermogravimetric analysis (TGA), and inductively coupled plasma optical emission spectrometry (ICP-OES). This approach enables traceable particle number concentration measurements of ligand-capped nonspheric particles with unknown chemical composition.

The advantageous electric, optical, magnetic, or catalytic properties of engineered nanomaterials (NMs) have led to their increasing production for applications in life and materials science, e.g., in medical diagnostics, optoelectronics, solid state lighting, energy storage and conversion, and catalysis.^1,2^ This fueled concerns regarding health and eco-toxicologic implications of NMs,^2,3^ see, e.g., REACH Annexes from the European Commission (EC).^4^ It also calls for validated protocols and methods for accurately and reliably characterizing NM properties relevant for their interaction with biological species and the environment like size, size distribution, shape, and surface chemistry. These needs the assessment of requirements, advantages, and limitations of methods providing size and particle concentration, and the establishment of nanoscale reference materials (nanoRMs)^5^ were addressed by many European research projects^6,7^ and interlaboratory comparisons (ILCs).^8−10^

Typical sizing methods are electron microscopy (EM), scattering-based methods like small-angle X-ray scattering (SAXS) and dynamic light scattering (DLS), and centrifugation-based methods such as analytical ultracentrifugation (AUC). EM measures dried individual particles on a solid support in vacuum, yielding two-dimensional (2D) information on three-dimensional (3D) nano-objects, and requires the evaluation of a statistically relevant number of particles. Scattering methods like SAXS read out ensembles of 10^6^–10^7^ particles in dispersion and face limitation for broad particle size distributions.^11^ Centrifugation-based sizing methods derive particle size from properties like the diffusion or sedimentation coefficient, assuming spheric particles. Thus, mean particle sizes can considerably differ between sizing methods, especially for nonspheric NMs.^12^ These results can be further biased by method-specific influences of sample preparation and data evaluation procedures.^13,14^ The latter is particularly relevant for the analysis of increasingly utilized SAXS measurements and of special relevance for the size and shape determination of nonspheric nano-object. The importance of accurate size and size distribution measurements also triggered the development of nanoRMs for instrument calibration and validation and standardization of sizing techniques by metrology institutes.^7,15^

At present, the vast majority of certified nanoRMs with sizes between 10 and 100 nm are spherical nanoparticles (NPs) such as gold, silica, and polystyrene NPs.^7,16^ Also in ILCs of sizing methods, mainly spheric NPs were measured.^10,17^ However, spheric nanoRMs are of limited value for establishing and calibrating size measurements of real-world NMs used in research and industrial applications, which can have irregular shapes and sizes. This metrological gap recently led to the release of first nonspheric nanoRMs with certified sizes like TiO_2_ nanorods and bimodal silica NPs from the Joint Research Centre of the European Commission (JRC; ERM-FD103 and ERM-FD102),^18^ and gold nanocubes from KRISS.^7^ Recently, BAM developed 8 nm cubic iron oxide NPs (IONPs) and certified their *area equivalent circular diameter* and *square edge length* derived from EM 2D-projection images according to ISO 17034:2017,^19^ and the ISO Guides 31:2015^20^ and 35:2017^21^ yielding BAM-N012.^22^ Like the spheric nanoRMs, these new nonspheric RMs are also exclusively suited as size standards. RMs for particle number concentration are even more rare. Presently, only a single quality control material is available consisting of 30 nm spheric gold particles (QC5050 from LGC).^23^

A concept for an efficient nanoRM development is multimethod nanoRMs applicable for several characterization methods. Ideal candidates for such a RM platform are lanthanide (Ln)-based NPs like NaYF_4_ or LiYF_4_ doped, e.g., with Yb^3+^ and Er^3^ or Tm^3+^. These upconversion NPs (UCNPs) (i). consist of multiple elements which do not naturally occur in most environments, (ii). can be reproducibly prepared in different sizes and shapes in relatively large quantities, (iii). with different chemical composition, and (iv). various surface coatings.^24^ Also, they are (v). chemically inert, and (vi). sufficiently long-term stable.^25^ (vii). Lanthanide ions can be sensitively detected and quantified with element-analytical methods like inductively coupled plasma-optical emission spectrometry (ICP-MS) and laser ablation-inductively coupled plasma mass spectrometry (LA-ICP-MS) and X-ray fluorescence methods like X-ray fluorescence computed tomography (XFCT) used, e.g., for quantitative bioimaging^26,27^ as well as with X-ray photoelectron spectroscopy (XPS).^28^ Also, luminescent Ln ions show several characteristic luminescence bands in the ultraviolet (UV), visible (vis), near-infrared (NIR), and short-wave infrared (SWIR), ideal for calibration purposes,^26,29^ and authentication barcodes.^30,31^

In the following, we present two monodisperse LiYF_4_:Yb,Tm bipyramids, UCNP-BP1 and UCNP-BP2, with peak-to-peak distances of (43 ± 2) nm and (29 ± 2) nm as new potential SI-traceable size and particle number concentration standards. The characterization of these NMs includes the determination of their size, shape, and concentration in cyclohexane dispersions by SAXS. The former measurements are supplemented by complementary EM studies, providing 2D information on UCNP size and shape parameters. The SAXS data on particle number concentration, complemented by gravimetry, thermogravimetric analysis (TGA), and inductively coupled plasma optical emission spectrometry (ICP-OES) measurements, yield mean particle density, chemical composition, and mass concentration values. These UCNP bipyramids can be utilized as test materials and tools for establishing and validating the sophisticated simulations required for determining the size and shape of nonspheric NMs by SAXS. Additionally, the photoluminescence spectra of the UCNPs are provided to underline their potential as multimethod RMs.

## Experimental Section

### Synthesis of UCNP Bipyramids UCNP-BP1 and UCNP-BP2

Yb,Tm-*co*-doped LiYF_4_ cores were prepared by adapting a thermal decomposition method established for the synthesis of monodisperse Yb,Er-*co*-doped NaYF_4_ UCNPs, exchanging NaOH for LiOH and Er^3+^ for Tm^3+^.^24,32^ as detailed in the Supporting Information (SI).

### Dynamic Light Scattering (DLS)

DLS measurements were performed on a Zetasizer Nano ZS (Malvern Instruments Ltd.).

### Inductively Coupled Plasma Optical Emission Spectroscopy (ICP-OES)

Quantification of Y, Yb, and Tm elemental concentration in the UCNPs by ICP-OES was performed with a SPECTRO Arcos-EOP (Model: FHX, 76004553) spectrometer. The calibration procedure is detailed in the SI.

### Thermogravimetric Analysis (TGA)

TGA studies were done with dried UCNPs obtained from the UCNP stock dispersions and a Hitachi STA 7200 setup with an AS3 Sample Charger.

### Transmission Electron Microscopy (TEM)

TEM images, obtained with a Talos F200S Microscope (Thermo Fisher Scientific) with an accelerating voltage of the electron beam of 200 kV, were analyzed with the software ImageJ. For the determination of the size parameters Fere*t*_max_ and Fere*t*_min_ from the 2D-projection images of the UCNP areas, 550 particles (UCNP-BP1) and 1600 particles (UCNP-BP2) were evaluated from 5 micrographs obtained with a pixel size of 123.2 pm. The size descriptors Fere*t*_max_ and Fere*t*_min_ were automatically measured and fitted with a Gaussian curve. The mean (μ) and standard deviation (σ_*x*_) of this curve were taken as the representative particle size of the sample. The angles between two adjacent planes were manually measured for 100 NPs using the angle tool. More details are given in the SI.

### Small-Angle X-ray Scattering (SAXS)

SAXS measurements were performed at the four-crystal monochromator beamline^33^ of the Physikalisch-Technische Bundesanstalt (PTB) at the synchrotron radiation source BESSY II in Berlin-Adlershof. For data analysis, the open-source Python extension “Computing Debye’s scattering formula for extraordinary formfactors” (CDEF)^34^ was employed to model the particle shape as a cloud of 30000 discrete point virtual scatterers with equal scattering potential *Z* and to calculate the corresponding scattering curve *I*(*q*) using the Debye equation. The fit of the measured data was performed by maximizing a log-likelihood function (objective function) assuming Gaussian distributed measurement uncertainties. For this maximation, a differential evolution algorithm was first applied to provide the starting point for the subsequent Markov chain Monte Carlo (MCMC) evaluation using the Python library “emcee”.^35^ More details are given in the SI.

### Steady-State Photoluminescence Measurements

Spectrally resolved measurements of the upconversion luminescence (UCL) of the UCNPs were performed on an Edinburgh Instruments Model FLS980-xD2-stm spectrofluorometer equipped with an 8 W 978 nm laser diode. All measurements were done with identical instrument settings at a defined excitation power density (*P*) of 38 W/cm^–1^.

## Results and Discussion

Lanthanide-based UCNPs, that consist of a host lattice such as an alkali metal lanthanide tetrafluorides AREF_4_ (A = Li^+^, Na^+^, K^+^) doped with pairs of sensitizer/activator ions like Yb/Er and Yb/Tm are utilized as reporters for bioimaging, sensing,^36^ nanotheranostics,^37,38^ optogenetics,^38^ anticounterfeiting, and barcoding,^30,39^ due to their ability to spectrally convert near-infrared (long-wavelength) light to short-wavelength photons (excitation power-density dependent UCL) and to show conventional down-shifted luminescence. Their potential as multimethod test and RM is, however, underexplored,^27^ despite their accessibility in different sizes and shapes with very narrow size distributions via reported protocols^40^ and upscaleable syntheses providing 2–5 g of NPs per batch.^32^ As a first set of nonspheric UCNP multiparameter and multimethod test materials and candidate nanoRMs for size and particle number concentration, we chose LiYF_4_:Yb,Tm UCNPs. The host material LiYF_4_ occurs only in the tetragonal phase at room temperature,^41^ yielding bipyramid-shaped NPs.^42^ Therefore, this host material was favored by us over hexagonal phased NaYF_4_-based UCNPs to complement our recently certified 8 nm IONP nanocubes.^22^

**Figure 1:**
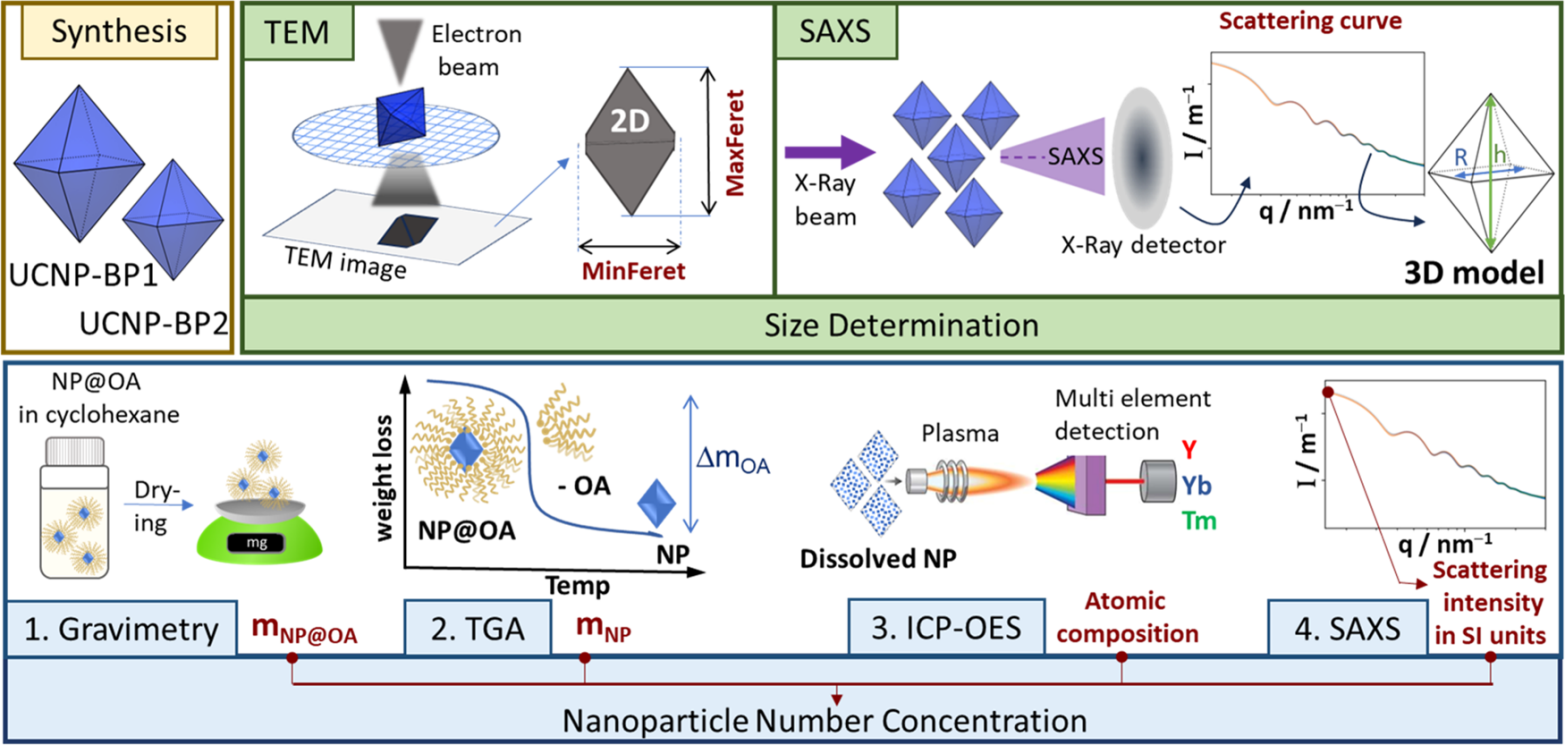
Overview of the characterization of the monodisperse LiYF_4_:Yb,Tm bipyramids including (i) synthesis, (ii) size determination by transmission electron microscopy (TEM) and small-angle X-ray scattering (SAXS), and (iii) particle number concentration determination by gravimetry, thermogravimetric analysis (TGA), and inductively coupled plasma optical emission spectroscopy (ICP-OES), and by SAXS.

For the preparation of the LiYF_4_:Yb,Tm bipyramid size and concentration standards UCNP-BP1 and UCNP-BP2, we used a thermal decomposition method yielding high-quality monodisperse UCNPs of controllable particle size.^32^ Prior to the metrologically traceable size and shape measurements of UCNP-BP1 and UCNP-BP2 with absolutely calibrated SAXS, supplemented by TEM, and the determination of the particle number concentration by SAXS, highlighted in Figure 1, UCNP synthesis was optimized regarding size and shape monodispersity of the resulting UCNP bipyramids by increasing the reaction time. Figure 1 also shows the SAXS concentration measurements with a combined input of gravimetry, TGA, and ICP-OES.

### Analytical Characterization of the UCNP Dispersions

**Figure 2:**
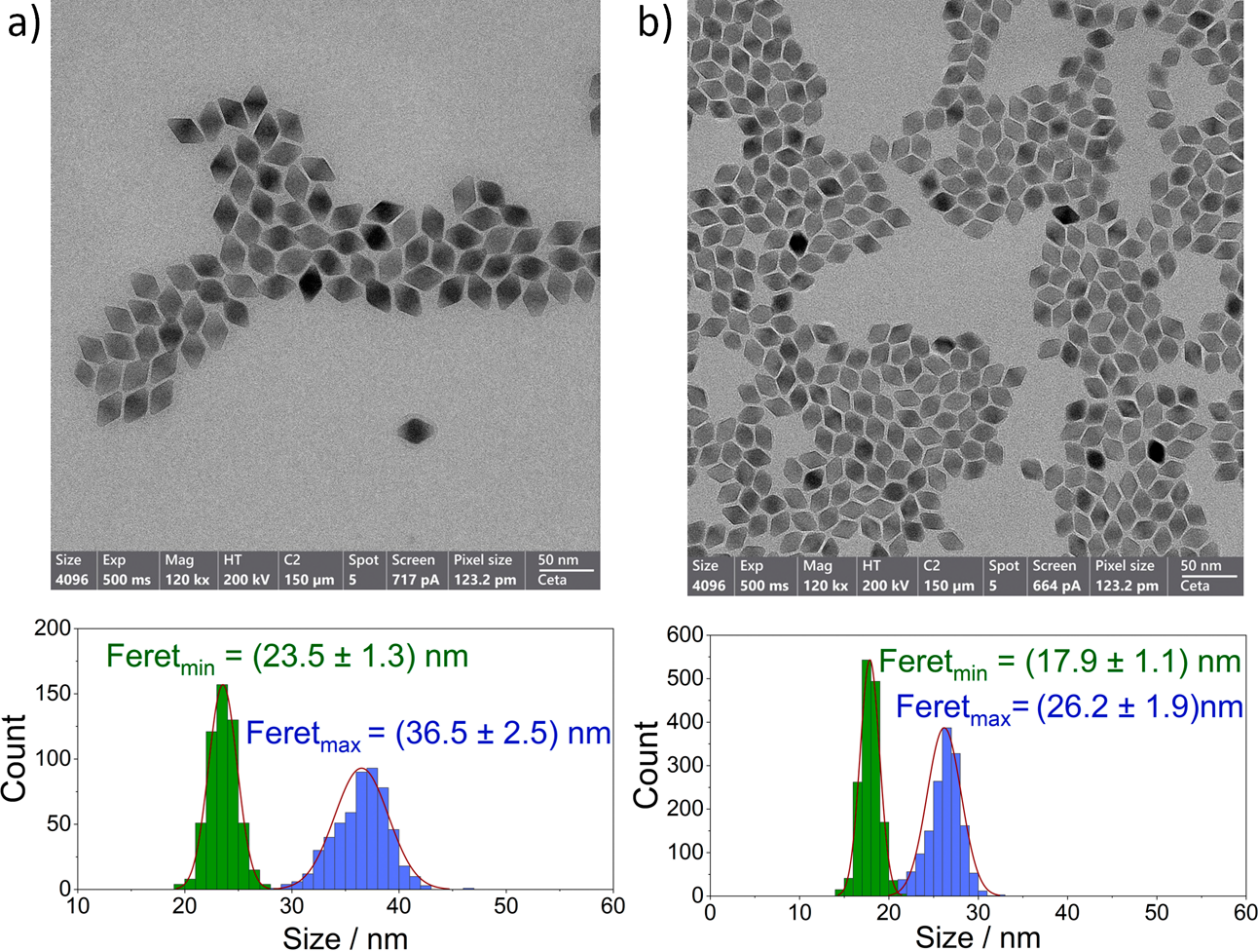
TEM images and histograms of Fere*t*_max_ (blue) and Fere*t*_min_ (green) of (a) UCNP-BP1 and (b) UCNP-BP2.

DLS measurements of dispersed UCNP yielded NP sizes of 21.0 and 15.7 nm (size by number) for UCNP-BP1 and UCNP-BP2 and confirmed the absence of large agglomerates or aggregates (SI, Figure S1). The TEM images revealed uniform bipyramidal shapes for both UCNPs and provided Fere*t*_max_/Fere*t*_min_ lengths of (36.5 ± 2.5) nm/(23.5 ± 1.3) nm and (26.2 ± 1.9) nm/(17.9 ± 1.1) nm for UCNP-BP1 and UCNP-BP2, respectively, with a dispersity below 10% as shown in Figure 2 and in Figures S3 and S4 in the SI. The angles between two adjacent planes, determined to be 59.7° (UCNP-BP1) and 60.4° (UCNP-BP2), were used to calculate the peak-to-peak distances from the Fere*t*_max_ values. This yielded values of (42.1 ± 3.5) nm and (30.3 ± 2.7) nm for UCNP-BP1 and UCNP-BP2. (SI, Figure S5).

For the determination of NP concentration of the UCNP stock dispersions, precisely known volumes of the dispersions were dried and subsequently weighed with a calibrated balance. This yielded mass concentrations of 48.4 and 21.4 mg/mL for UCNP-BP1 and UCNP-BP2, respectively. The dried solid UCNPs were then analyzed by TGA to allow for the consideration of the contribution of the ligand mass to the particle mass. TGA measurements provided an overall mass loss of (21.3 ± 0.6) wt % and (22.4 ± 1.0) wt % for UCNP-BP1 and UCNP-BP2, due to the loss of the oleate surface ligands at elevated temperatures of 200–480 °C. These values were then used for the calculation of the UCNP mass concentrations, yielding values of 38.0 and 16.4 mg/mL for UCNP-BP1 and UCNP-BP2, respectively. These data are used as complementary input for the determination of the particle number concentrations by SAXS described in the following section.

### SAXS Measurements

SAXS, which yields number-weighted particle size distributions, is a well-recognized method for characterizing the size and particle number concentration of dispersed NPs.^43^ It fulfills the requirements of the EC recommendation (2022/C 229/01) for the definition of NMs^44^ and is recommended for the determination of these quantities by ISO 17867:2020 and ISO 23484:2023.

### Traceable SAXS Size Measurements

The development of certified RMs (CRMs)^22^ requires the characterization with analytical methods traceable to the International System of Units, such as the meter for size. For particle size measurements with SAXS, this is achieved by the calibration of the X-ray photon energy, the detector pixel size, and the sample-to-detector distance. For the PTB setup, the detector pixel size and the calibration of the photon energy have been previously determined in a traceable way.^45^ The sample-to-detector distance is obtained by triangulation for each measurement using absolute optical encoders and a sample with strong scattering features such as mesoporous silicon dioxide SBA-15 or silver behenate. Thereby, the one-dimensional (1D) scattering curves are established as functions of the inverse length scale in m^–1^.

**Figure 3:**
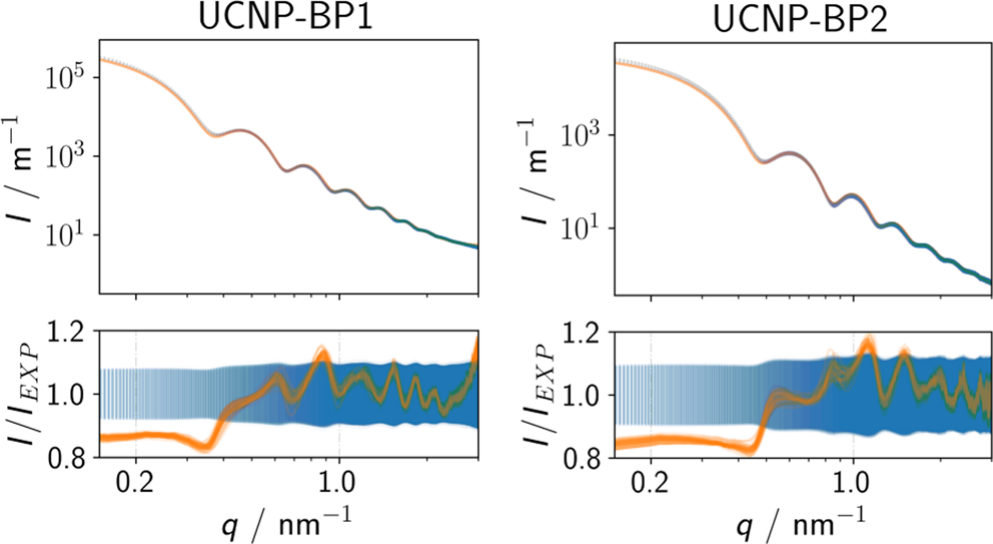
SAXS scattering curves show the scattered intensity *I* as a function of momentum transfer *q*. The measured data *I*_EXP_ are shown as blue bars, indicating the experimental uncertainties, while the fits of the SAXS curves are given in orange.

The scattering curves of cyclohexane dispersions of UCNP-BP1 and UCNP-BP2 as measured by SAXS are shown in Figure 3 as blue bars indicating the experimental uncertainties. These data were fitted with a bipyramidal particle model. In this model, the edge length *R* with a number-weighted log-normal size distribution *L*(*R*, σ) and the height of the bipyramid *h*, i.e., the peak-to-peak distance, are regarded as independent (SI). As for organic ligand molecules like oleic acid, the ligand shell is normally not detected by SAXS, a simple core model was utilized for the fits of the measured SAXS curves and not a core–shell model. The fits are shown in Figure 3 as orange curves, each representing an individual set of fit parameters from the posterior probability distribution. This is used to determine an uncertainty estimate for each parameter. Within these uncertainties, the particle model provides a plausible description of the measured data of UCNP- BP1 and UCNP-BP2 with reference to Figure 3. The slightly larger deviations between the measured data and the fits in the Guinier region could possibly indicate a small contribution from agglomerated particles. The size parameters or dimensions of UCNP-BP1 and UCNP-BP2 derived from the fits of the SAXS scattering curves, which are summarized in Table 1, agree with the results of the TEM measurement within the stated uncertainties.

**Table 1:** SAXS Results of the Number-Weighted Size Distribution of UCNP-BP1 and UCN-BP2 with the Square Base and Lognormally Distributed Edge length *R*, the Standard Deviation σ, and Height *h*^a^

	*R*/nm	σ/nm	*h*/nm	*h*/nm
sample	SAXS	SAXS	SAXS	TEM
UCNP-BP1	23.6 ± 0.5 (2.1%)	1.5 ± 0.2 (13.3%)	43 ± 2 (4.6%)	42.1 ± 3.5
UCNP-BP2	19.0 ± 0.5 (2.6%)	1.23 ± 0.09 (7.3%)	29 ± 2 (6.9%)	30.3 ± 2.7

a The uncertainties are provided as a 1σ confidence interval. The percentage values given in brackets represent relative uncertainties.

### Traceable Measurement of UCNP Particle Number Concentration

For traceable particle concentration measurements with SAXS, additionally, the quantum efficiency of the X-ray detector and the responsivity of the photodiodes, measuring the incoming photon flux, must be known. This is achieved by comparison to the PTB’s primary radiation detector standard, a cryogenic electrical substitution radiometer.^46^

The reliability of particle number concentration *C* measurements by SAXS has been confirmed in an ILC of different analytical methods on the determination of the particle number concentration of dispersed spheric gold NPs.^23^ However, until now, SAXS has been solely utilized for concentration measurements of spheric NPs in dispersion,^23^ but not for concentration measurements of NMs with a more complex shape as revealed by our UCNP bipyramids. Prerequisites for traceable particle concentration measurements with SAXS are (i). a fully calibrated experimental setup for the measurement of the differential scattering cross-section per volume and (ii). a known sample thickness. This together allows the measurement of the momentum transfer and the scattering intensity in the International System of Units. Also, (iii). a model for the particle shape and size-distribution is required and (iv). the electron density contrast (Δρ_e_) between the particles and the solvent must be known.^47^ The latter was calculated from the mass concentration *c*_m_ of the UCNPs in the dispersions measured by SAXS as detailed in the SI (see eq S5),^47^ utilizing the ratio of the NP constituents Y, Yb, and Tm determined by ICP-OES and considering the mass contributions of the organic surface ligands derived from TGA. In the case of UCNP-BP1 and UCNP-BP2, Δρ_e_ was calculated from the mass concentration *c*_m_ of the UCNPs in the dispersions measured by SAXS as detailed in the SI (see eq S5).^47^ For the calculation of the number concentration *C*, a mass concentration of both samples of *c*_m_ = 5 mg/mL, corrected by the mass change of (21.3 ± 0.6) wt % (UCNP-BP1) or (22.4 ± 1.0) wt % (UCNP-BP2) determined by TGA due to the oleic acid ligands, was assumed. The effective electron number *Z* results from the atomic form factors of the respective elements at a photon energy of 8 keV, taken from the database of the National Institute of Standards and Technology (NIST).^48^ The particle number concentration *C* of the UCNPs can be calculated from the SAXS measurements with an estimated uncertainty of less than 17% according to the results shown in Table 2. Also, the previously unknown mass density **ρ**_**m**_ of the Yb,Tm-doped LiYF_4_ bipyramids was calculated from the results of the particle number concentration *C* (SI, eq S1). This yields compatible values for both samples.

**Table 2:** Particle Number Concentration *C* and Mass Density ρ_m_ of the UCNPs Derived from SAXS^a^

sample	*c*_m_ (10^–24^ g/cm^3^)	*V* (10^3^ nm^3^)	*Z*/1	*M* (g/mol)	*C* (10^14^ cm^–3^)	ρ_m_ (g/cm^3^)
UCNP-BP1	3.935	8.0 ± 0.2	75.5 ± 0.3	172.08 ± 0.03	1.3 ± 0.2	3.9 ± 0.5
UCNP-BP2	3.88	3.52 ± 0.09	75.2 ± 0.2	172.10 ± 0.03	2.4 ± 0.4	4.7 ± 0.7

a The calculations of the molar mass *M* and the effective electron number *Z* are based on the results of the ICP-OES measurements of the elemental composition of UCNP-BP1 and UCNP-BP2 and the uncertainties present relative standard deviations. The calculated uncertainties of the concentration *C* and the mass density ρ_m_ represent estimates.

### Stability Studies

An application-relevant property of every test and RM presents its stability under defined storage conditions. For CRM, stability data are always included in the uncertainty budgets of the certified property.^22^ According to our experience with different types of ligand-capped UCNPs, varying in size, shape, and surface ligands, oleate-capped UCNPs dispersed in cyclohexane are commonly colloidally stable for up to 2 years when stored in the refrigerator at 4 °C. For the production and supply of UCNP-based test and RM intended in the future, we performed first stability screening tests with SAXS and TEM of cyclohexane dispersion of oleate capped UCNP-BP1 and UCNP-BP2 with NP concentrations of *c* = 48.4 and 21.4 mg/mL, kept at 4 °C in the refrigerator. These UCNP dispersions were examined 15 months after UCNP synthesis with TEM. As follows from the TEM images and the corresponding histograms shown in the SI (Figures S3 and S4), there seems to be a trend pointing to a slight increase in the size descriptors Fere*t*_max_ and Fere*t*_min_, but this trend is not significant. UCNP stability was also examined by SAXS, here after 8 months. These measurements did not reveal a hint for changes in the size and shape of UCNP-B1 and UCNP-B2 as well as for changes in the UCNP concentration of the dispersions (SI, Figure S5). Based on these first screening studies and our previous experiences with similar NMs, we assume that UCNP-BP1 and UCNP-BP2 are stable for at least 15 months, most likely even for 2 years under these storage conditions. In the future, more systematic stability studies are planned as has been performed for the iron oxide nanocubes BAM-N012,^22^ which are, however, CRMs.

### Photoluminescence Studies

To highlight the potential of our UCNPs as multimethod test and RM, the spectrally corrected UCL spectra of UCNP-BP1 and UCNP-BP2 dispersed in cyclo hexane were determined utilizing multiphoton excitation at 980 nm. The emission spectra of UCNP-BP1 and UCNP-BP2, depicted in Figure S6 in the SI, reveal the characteristic Tm^3+^ emission bands in the UV (340–360 nm), blue (450–416 nm), and red (635–680 nm) wavelength regions and the expected dependence of UCL intensity and color on UCNP size.^48^ Please note that the UCL efficiency of LiYF_4_-based UCNPs is known to be inferior to that of NaYF_4_-based UCNPs and especially to core/shell UCNPs with thick surface protection shells.^49^ Such photoluminescence data could possibly be utilized in the future for controlling the calibration and performance of spectro fluorometers by comparing the intensity ratios of the different Ln ion emission bands as exploited for our certified Ln-based multiemitter glass BAM-F012.^29^ In addition, such UCL measurements with UCNP-based RMs containing common sensitizer/activator pairs such as Yb/Tm and Yb/Er could in the future pave the road to comparable and even standardized measurements of P-dependent UCL efficiencies or quantum yields of UCNPs requiring UCL standards with known P-dependencies of their UCL spectra, quantum yields, and decay kinetics.

## Conclusions and Outlook

In summary, we characterized two nanoscale, monodisperse, multicolor emissive LiYF_4_:Yb,Tm bipyramids, UCNP-BP1 and UCNP-BP2, focusing on small angle X-ray scattering (SAXS) measurements with an absolutely calibrated SAXS setup. UCNP-BP1 and UCNP-BP2, with peak-to-peak distances of (43 ± 2) nm and (29 ± 2) nm, are intended for use as nonspheric multimethod test materials and candidate reference materials (RMs).

Such bipyramids, which can be obtained from BAM upon request, can be used for sizing methods such as SAXS, to establish and validate the sophisticated simulations required for the determination of the size and shape of nonspheric NMs by SAXS and comparing different data fitting routines/algorithms used SAXS data evaluation. In addition to the determination of the size and shape of these bipyramids in cyclohexane dispersions with SAXS, complemented by transmission electron microscopy (TEM) for 2D size information, we demonstrated the determination of the particle number concentration of these NM dispersions by absolutely calibrated SAXS. The latter included the complementary input from gravimetry, thermogravimetric analysis (TGA), and inductively coupled plasma optical emission spectrometry (ICP-OES) measurements. Our approach enables to measure the particle number concentration and density of ligand-capped nonspheric particles with unknown chemical composition, urgently needed for number-concentration measurements of complex real-world nanoparticles. Future validation of this approach will require an interlaboratory comparison (ILC) with other SI-traceable methods like spICP-MS.

Overall, our monodisperse multielement lanthanide (Ln)-based NMs present novel multimethod test materials, RM candidates, and calibration tools for a variety of methods used in NM characterization and imaging. This multimodality originates from the tuneability of the size, shape, chemical composition, and surface chemistry of this class of NMs and the unique optical and magnetic properties of many Ln ions. The Ln-based upconversion nanoparticles (UCNPs) presented here, constituting of different luminescent lanthanide ions, can be, e.g., sensitively measured, and quantified by analytical methods such as LA-ICP-MS, XRF, XFCT, and LIBS, and utilized for XPS studies. Moreover, the characteristic photoluminescence of these Ln-based NMs can be exploited for the calibration and performance control of photoluminescence measuring devices as shown by us for our certified Ln-based fluorescence standard BAM-F012.

In the future, our concept of multimethod NMs can speed up the development, production, and supply of urgently needed, well characterized test NMs and nanoRMs for analytical methods utilized for NM characterization. This approach can facilitate correlative measurements with different sizing and imaging methods, relying on different mechanisms of signal generation and detection. Based on the results of this study and our expertise in producing and certifying nanoscale and optical RMs, and preparing and characterizing UCNPs, we will expand our nanoRM platform of iron oxide NMs to NMs of different size, shape, and chemical composition with varying capping ligands as well as to core–shell structures. Additionally, reference data for selected methods will be provided to ensure the accuracy, comparability, standardization, and compatibility of characterization methods for complex NMs. For example, the reference data, that will be provided by us based on this study and ongoing studies of other NMs, can be utilized to establish and validate sophisticated data simulations as required for determining the size and shape of nonspheric NMs by SAXS or for the feeding of deep learning approaches.^50^

## Supporting Information Available

Additional experimental details for material synthesis and all characterization methods; stability studies by TEM and SAXS; ICP-OES and TGA data; 3D model of a bipyramid; photophysical studies, as well as the detailed results of the size and particle number concentration measurements by SAXS and the corresponding calculations (PDF)
