# Supplementary material for: Adding More Shape to Nanoscale Reference Materials—LiYF4:Yb,Tm Bipyramids as Standards for Sizing Methods and Particle Number Concentration
Source: Anal Chem. 2024 Nov 13;96(48):19004–11. doi: 10.1021/acs.analchem.4c03641 (PMC11618739; doi:10.1021/acs.analchem.4c03641)
Supplement: Supplementary file 1 — ac4c03641_si_001.pdf [file ac4c03641_si_001.pdf]

Supporting Information (SI)

Adding More Shape to Nanoscale Reference Materials – $LiYF_4$:Yb,Tm Bipyramides as Standards for Sizing Methods and Particle Number Concentration

Jérôme Deumer[1,#], Elina Andresen[2,#,*], Christian Gollwitzer[1], Robin Schürmann[1,*], and Ute Resch-Genger[2,*]

[1]Physikalisch-Technische Bundesanstalt, Abbestraße 2-12, 10587, Berlin, Germany;

email: robin.schuermann@ptb.de

[2]Division *Biophotonics*, Bundesanstalt für Materialforschung und -prüfung, Richard-Willstaetter-Straße 11, 12489 Berlin, Germany; email: elina.andresen@bam.de; ute.resch@bam.de

#: both authors contributed equally

1. Experimental information	2
2. Synthesis of Lanthanide-based Upconversion Nanoparticles UCNP-BP1 and UCNP-BP2	3
3. Analytical Characterization of UCNP-BP1 and UCNP-BP2	
3.1 Inductively Coupled Plasma Optical Emission Spectroscopy (ICP-OES)	4
3.2 Dynamic Light Scattering (DLS)	4
3.3 Thermogravimetric Analysis (TGA)	5
3.4. Electron Microscopy and Stability Studies	6-8
3.5. Photoluminescence Studies	9
4. SAXS Measurements	
4.1. Size Distribution	10
4.2. Particle Number Concentration	11
5. References	19

1. Experimental Information

Transmission Electron Microscopy (TEM).

TEM images were obtained with a Talos F200S Microscope (Thermo Fisher Scientific) with an accelerating voltage of the electron beam of 200 kV. The samples were prepared by drop casting of diluted UCNP dispersions (1 mg/mL UCNP in cyclohexane) onto a 3 mm copper grid (lacey, 400 mesh). The grids were allowed to dry under air at room temperature (rt), yielding monolayers of well separated particles on the TEM grids. The obtained micrographs were analyzed with the software ImageJ. For the determination of the size parameters $Feret_{max}$ and $Feret_{min}$ from the 2D-projection images of the UCNP areas, 550 particles (UCNP-BP1) and 1600 particles (UCNP-BP2) from 5 micrographs obtained with 120kx magnification were evaluated. This number matches the minimum number recommended, for data evaluation, the size distribution descriptors (e.g., $Feret_{max}$ and $Feret_{min}$) were automatically measured after background subtraction (rolling ball radius = 500 pixels, light background), using a Gaussian Blur filter with sigma (radius) = 3 and manual adjustment of the threshold. The obtained UCNP diameters were plotted in the form of a histogram which was subsequently fitted with a Gaussian curve. The mean (μ) and standard deviation (σ_x) of this curve were taken as the representative particle size of the sample. The angle between two adjacent planes was manually measured for 100 NPs using the angle tool in Image J.

Small-angle X-ray scattering (SAXS).

Since the SAXS experiments were performed in vacuum, the diluted colloidal solutions of the UCNP-BP1 and -BP2 samples were filled into borosilicate glass capillaries with a homogeneous thickness and sealed vacuum-tight before the measurement. The sample was then loaded into the experimental vacuum chamber, which is connected to the four-crystal monochromator (FCM) beamline of the PTB laboratory at the synchrotron radiation facility BESSY II in Berlin. For the experiment, synchrotron radiation is generated by a bending magnet, monochromatized and directed through the beam line to the sample holder, producing a thin X-ray beam with a cross-sectional area of about $150\ \mu m$ high and $400\ \mu m$ wide at the sample position. The FCM beamline enables experiments in a wide range of photon energies from 1.75 keV to 10 keV [1]. All SAXS experiments were performed at 8 keV with Si(111) monochromator crystals with a spectral resolving power of $E/\Delta E = 10^4$ and a photon flux in the range of $\Phi \simeq 10^{10} s^{-1}$..[1] $p = 172\ \mu m$[OBJ].[2] The use of a Synchrotron Radiation Electrical Substitution Radiometer (SYRES) as primary detector standard allows the determination of the radiant power in absolute units and thus allows the determination of the quantum efficiency of all photodiodes in the experimental setup and the PILATUS1M detector.

Dynamic light scattering (DLS). Dynamic light scattering (DLS) measurements were performed with a Zetasizer Nano ZS (Malvern Instruments Ltd.) equipped with a 633 nm laser (4 mW) in backscattering mode (scattering angle 173°) using 1 cm disposable cuvettes. The data was analyzed by the implemented Zetasizer Nano software (version 7.13) that uses the cumulant method and the Malvern General Purpose Non-Negative Least Squares (NNLS) fitting for the analysis of the intensity correlation function.

Inductively coupled plasma optical emission spectroscopy (ICP-OES). The ICP standard solutions (1000 mg/L in nitric acid (2-3%)) used for the calibration required for the quantification were purchased from Sigma Aldrich. The calibration was performed with 10 standard solutions covering the range of 0-2500 µg/L, 0-80 µg/L and 0-7200 µg/L for Yb^{3+}, Tm^{3+} and Y^{3+}, respectively. The samples were dried, dissolved in nitric acid, and further diluted in milliQ water prior the measurements.

All given volumes were measured by calibrated pipettes from Eppendorf. Dilutions were prepared in class A volumetric flasks. Gravimetric measurements of the dried samples were performed on a calibrated balance BP211D (0.0001 g-210.0 g, d = 0.01 mg, e=0.1 mg) from Sartorius AG.

Thermogravimetric analysis (TGA). TGA measurements were performed with dried solid UCNPs utilizing a Hitachi STA 7200 set-up with an AS3 Sample Charger over a temperature range of 30-600 °C under nitrogen flow (200 ml/min) with a heating rate of 10 °C/min.

Stability screening. First screening studies of the stability of the cyclohexane dispersions of UCNP-BP1 and UCNP-BP2 over 15 months were performed by TEM for sample storage at 4°C and concentrations of UCNP-BP1 and UCNP-BP2 of c = 48.4 mg/mL and 21.4 mg/mL, respectively. Thereby, a TEM sample from the stock NP-dispersion was prepared, measured, and evaluated as for the freshly prepared samples.

.

2. Synthesis of Lanthanide-based Upconversion Nanoparticles UCNP-BP1 and UCNP-BP2

Synthesis of the $LiYF_4$:Yb,Tm nanoparticles UCNP-BP1 and UCNP-BP2. In a typical procedure, a methanolic solution of defined amounts of $RECl_3 \cdot 6H_2O$ (RE = Y, Yb, Tm) precursors (total 5 mmol, ratio Y:Yb:Tm = 79.5:20:0.5 for $LiYF_4$:Yb^{3+},Tm^{3+}) was added to a mixture of oleic acid (OA) and 1-octadecene (ODE) in a 250 mL three-necked flask. The exact amount of the reagents and the reaction conditions are provided in Table S1. The stirred reaction mixture was heated to 150 °C for 30 min under an argon flow, then vacuum was applied for further 30 min at 150 °C to remove remaining water, leading to a transparent yellow solution. The reaction mixture, containing the lanthanide precursors, was then cooled down to room temperature under a constant argon flow. Subsequently, a methanolic solution (30 mL) containing LiOH and NH_4F was added, and the resulting suspension was heated to 120 °C for 30 min to remove excess methanol. Subsequently, the reaction mixture was heated to 325 °C under reflux under a gentle argon flow, kept at this temperature for a reaction period of 30 min (UCNP-BP2) or 60 min (UCNP-BP1), and then cooled down to room temperature. The resulting core UCNPs were purified by precipitation with ethanol and collected by centrifugation (10 min, 5000 rpm). The obtained UCNPs were further re-dispersed in chloroform, precipitated with ethanol, and collected by centrifugation (10 min, 5000 rpm). Then, the UCNPs were re-dispersed in cyclohexane, precipitated with acetone, and collected by centrifugation (10 min, 5000 rpm). Finally, the UCNP-BP1 and UCNP-BP2 were dispersed in cyclohexane and stored at 4 °C.

Table S1: Synthesis of Yb, Tm - co-doped core nanoparticles UCNP-BP1 and UCNP-BP2 ($LiYF_4$: 20 % Yb, 0.5 % Tm)

	$YCl_3 \cdot 6H_2O$	$YbCl_3 \cdot 6H_2O$	$TmCl_3 \cdot 6H_2O$	NH_4F	LiOH	Oleic acid	1-Octa-decene	React. time
	mg (mmol)	mg (mmol)	mg (mmol)	mg (mmol)	mg (mmol)	mL	mL	min
**UCNP-BP1**	1205.9 (3.98)	387.5 (1.00)	7.5 (0.03)	741.5 (20)	311.4 (13)	50	75	60
**UCNP-BP2**	1205.3 (3.97)	387.7 (1.00)	7.2 (0.03)	740.5 (20)	311.3 (13)	50	75	30

3. Analytical Characterization of UCNP-BP1 and UCNP-BP2

3.1. Inductively Coupled Plasma Optical Emission Spectroscopy (ICP-OES)

Table S2: Material composition (atomic concentration) of the UCNP-BP1 and UCNP-BP2 determined by ICP-OES measurements.

Sample (conc. of stock solution)	**m** (UCNP@OA) mg	c (dissolved in HNO3) mg/mL	Measured by ICP-OES		
			Tm µg/L	Y µg/L	Yb µg/L
UCNP-BP1 (c = 48.4 mg/ml)	4.84 ± 0.03	3.2	24.33 ± 0.14	3045 ± 14	1368.5 ± 6.7
UCNP-BP2 (c = 21.4 mg/ml)	2.14 ± 0.03	1.5	13.15 ± 0.18	1369 ± 16	666.7 ± 7.6

3.2. Dynamic Light Scattering (DLS)

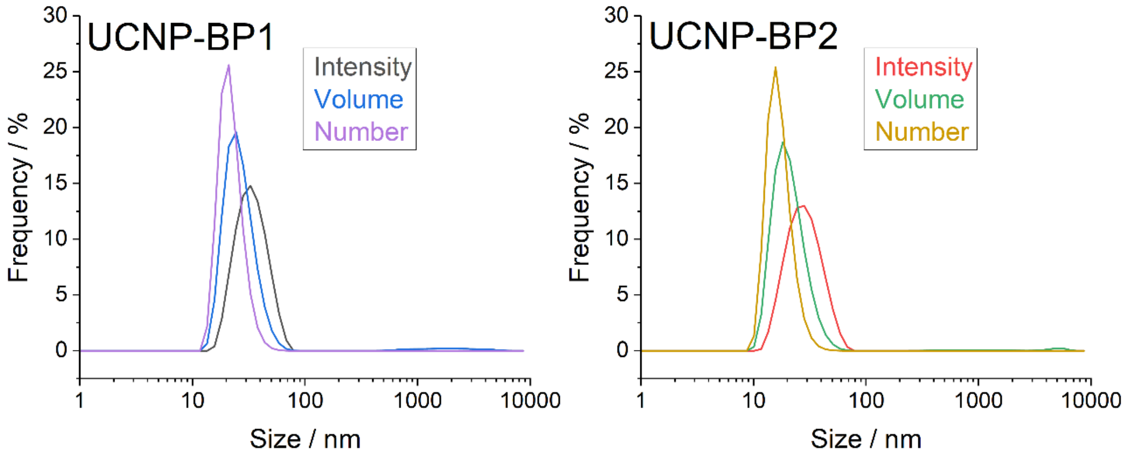

*Figure S1: DLS measurements of diluted dispersions of UCNP-BP**1** and UCNP-BP**2** in cyclohexane (c = 1 mg/mL) using the MALVERN General Purpose NNLS algorithm.*

3.3. Thermogravimetric analysis (TGA)

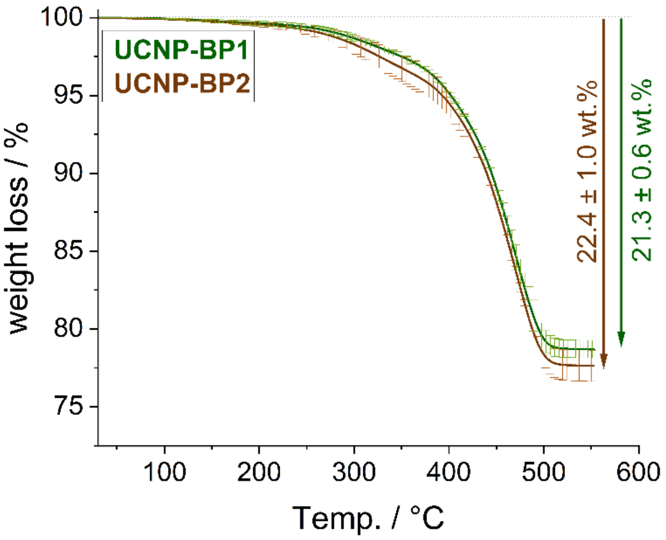

Figure S2: Thermogravimetric analysis (TGA) curves of UCNP-BP1 and UCNP-BP2 (triplicate measurement). The weight loss in the temperature range of 200°C-480°C is attributed to the loss of the oleic acid surface ligands.

3.4. Electron Microscopy (EM) and Stability Studies

UCNP-BP 1

As-synthesized

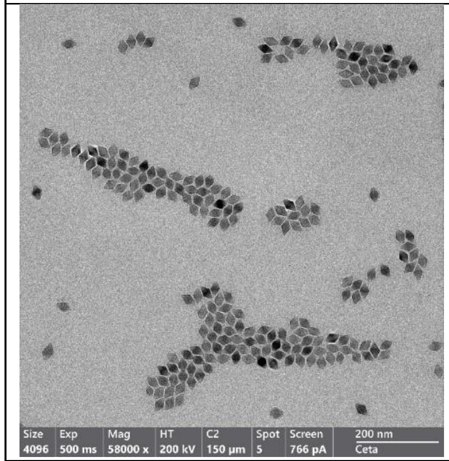

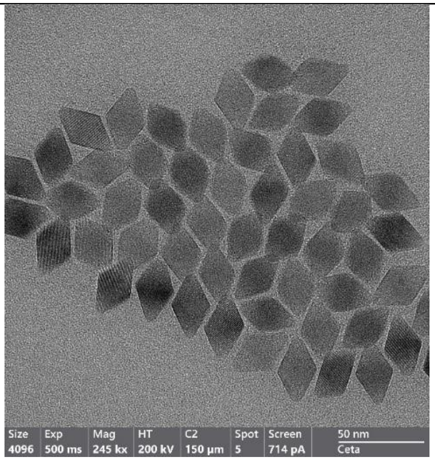

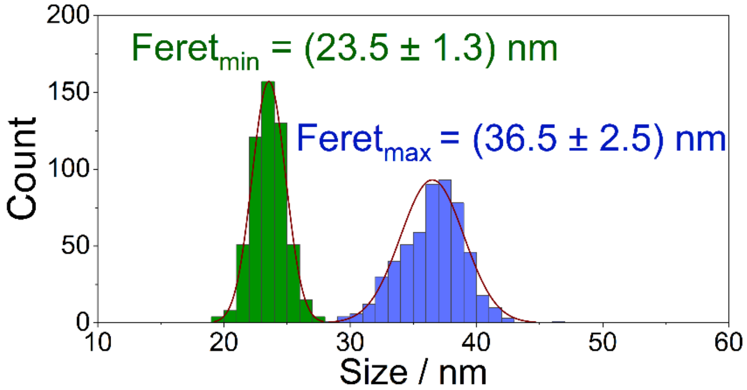

After 15 months

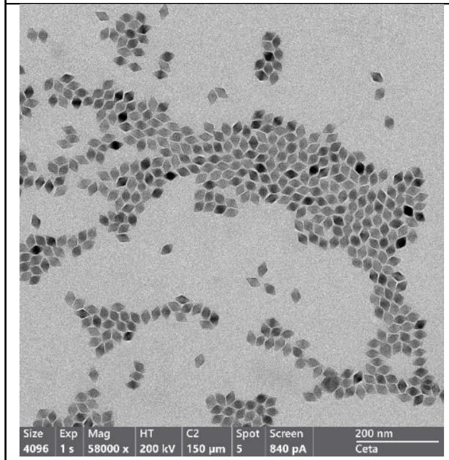

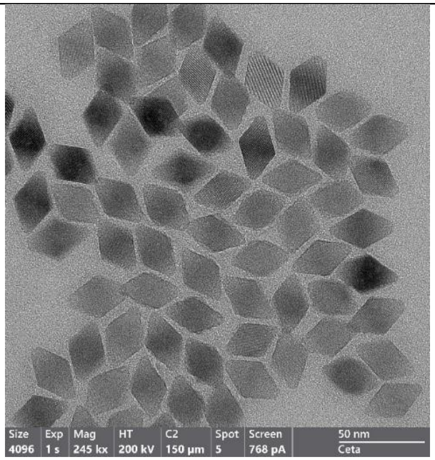

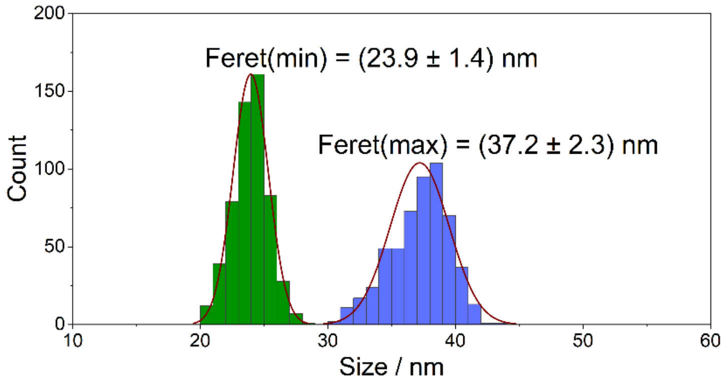

Figure S3: TEM images and histograms of freshly prepared samples **UCNP-BP1** and after storage at 4°C for 15 months at 120 kx (left panel) and 245 kx (middle panel) and the corresponding histograms for the descriptors Feret(max) / Feret(min) (right panel).

UCNP-BP 2

As-synthesized

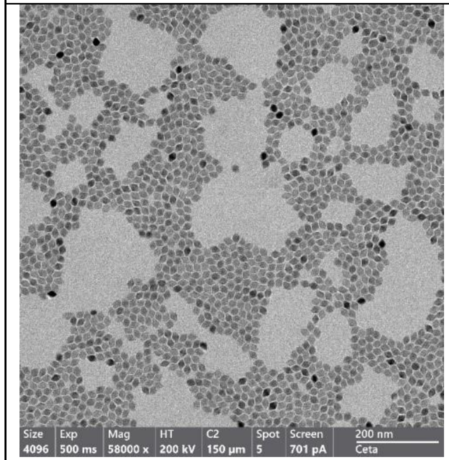

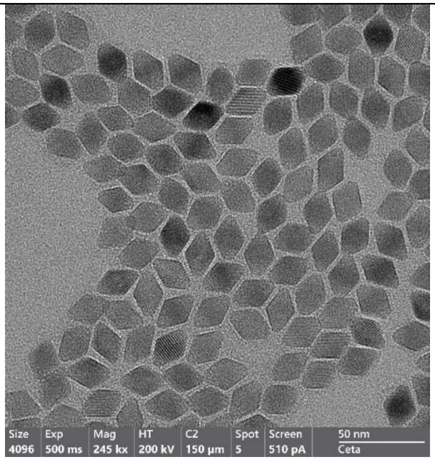

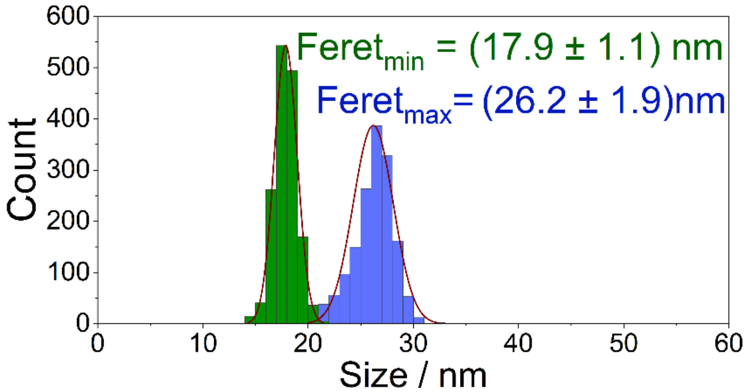

After 15 months

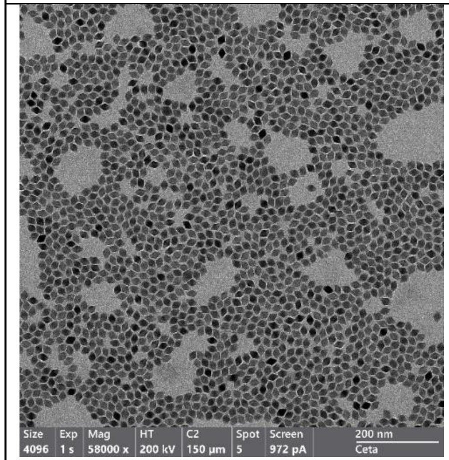

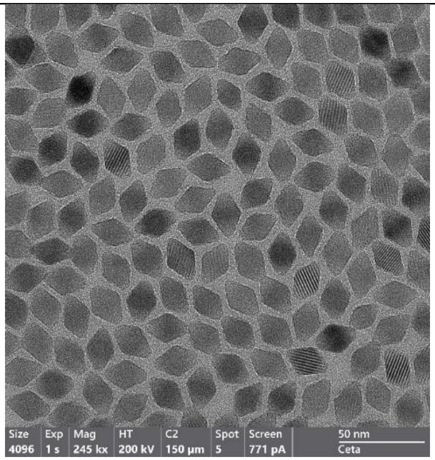

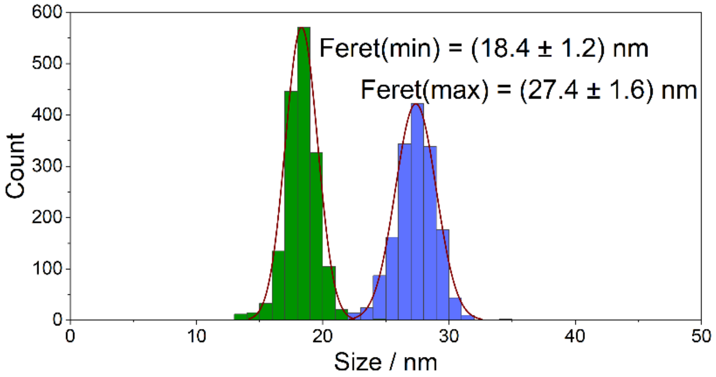

Figure S4: TEM images and histograms of freshly prepared samples ***UCNP-BP2*** *and after storage at 4°C for 15 months at 120 kx (left panel) and 245 kx (middle panel) and the corresponding histograms for the descriptors Feret(max) / Feret(min) (right panel).*

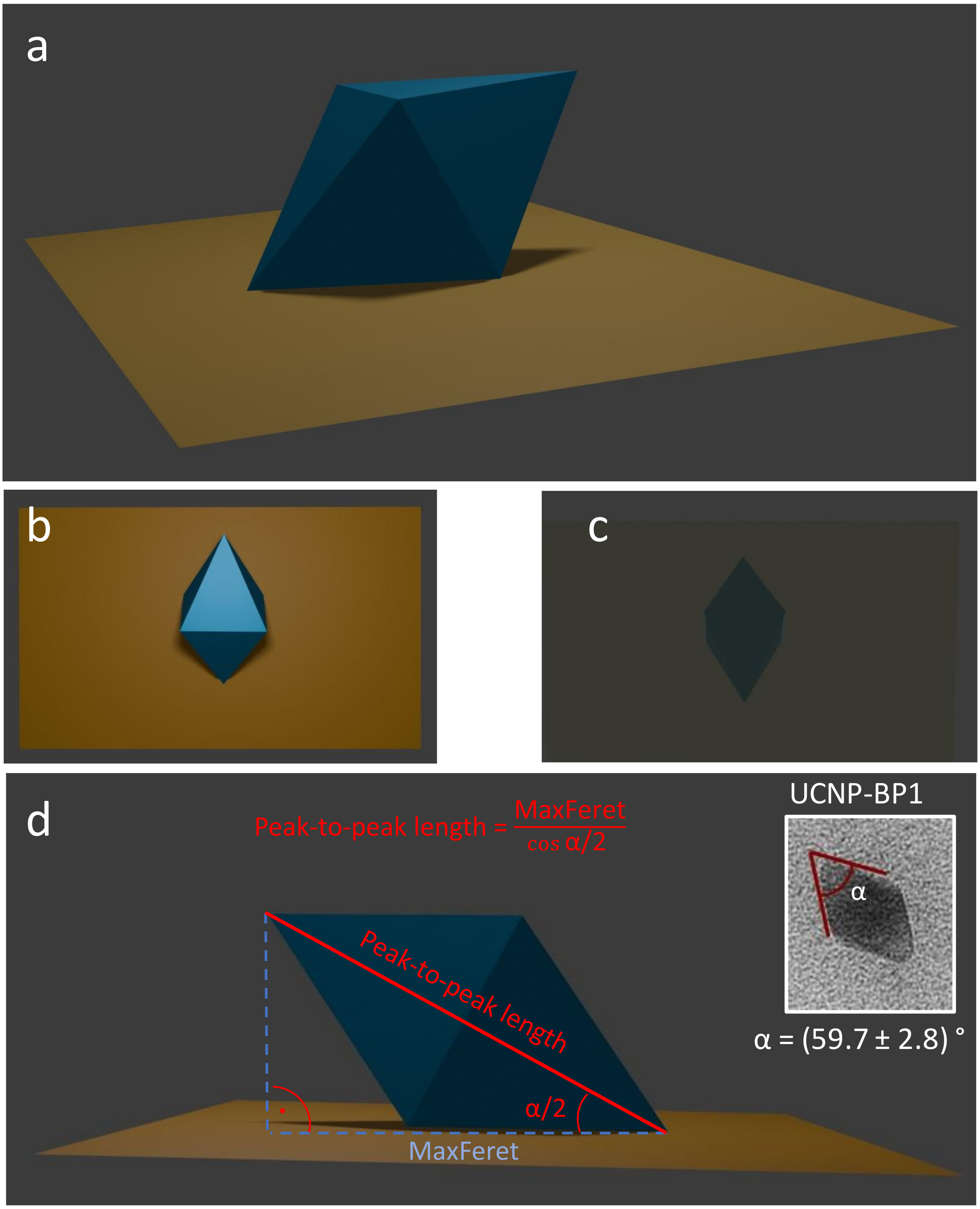

*Figure S5: a) 3D model of an UCNP bipyramid on a TEM grid showing b) a top view, c) a bottom view, and d) a side view, illustrating the calculation of the peak-to-peak distance of **UCNP-BP1** and **UCNP-BP2** from the Feret$_{max}$ value and the angle between the adjacent planes as determined by TEM. The inset shows a TEM micrograph of one **UCNP-BP1** nanoparticle with a marked angle (α ± std)° determined from 100 NPs. The corresponding value for **UCNP-BP2** is α = (60.4 ± 3.4)°.*

3.5. Photoluminescence Studies

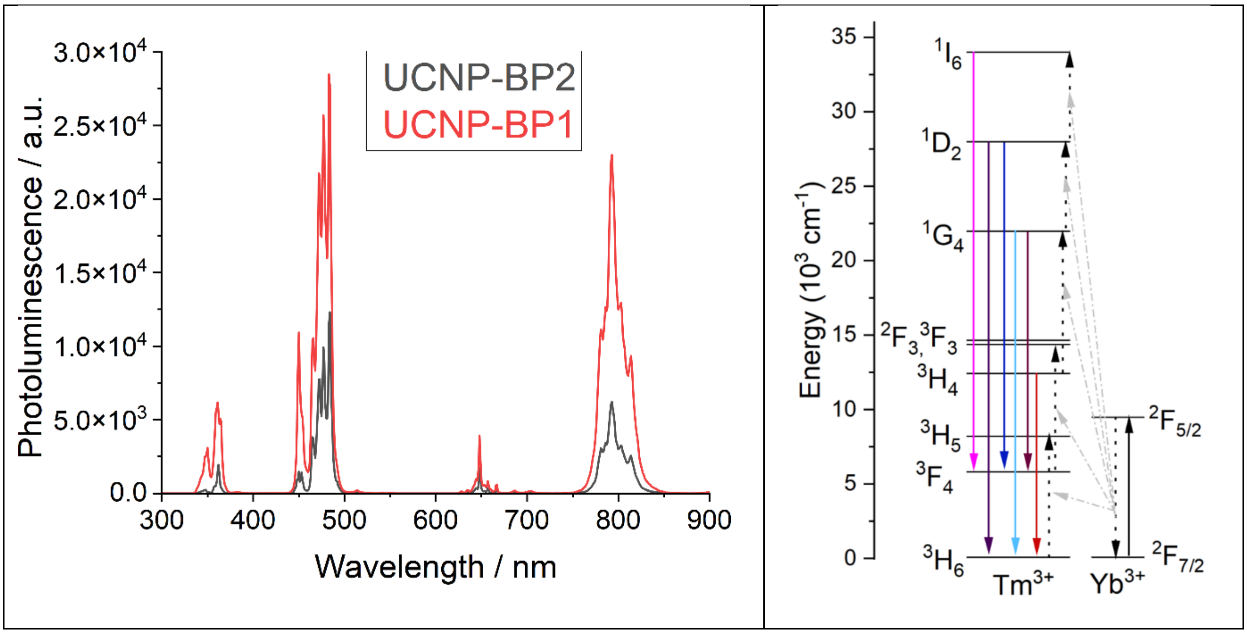

Figure S6: Left: Spectrally corrected steady-state photoluminescence spectra of oleate capped UCNP-BP1 and UCNP-BP2 dispersed in cyclohexane (1 mg/mL) under 980 nm excitation (38 W/cm^2) provided in relative photoluminescence intensities (left). Both spectra were obtained under identical, precisely known measurement conditions including the same excitation power density (P) of 38 W/cm^{-1}. Right: Corresponding energy diagram of Tm^{3+} and Yb^{3+} depicting the origin of the upconversion (UC) luminescence bands. This energy diagram summarizes the population and depopulation pathways of the Tm^{3+} energy levels, accounting for the differently colored Tm^{3+} emission bands. Briefly, absorption of two or more NIR photons by the Yb^{3+} ions results in an energy transfer upconversion (ETU) process from its excited $^2F_{5/2}$ level to different energy levels of the Tm^{3+} ions. Radiative relaxation of the electronically excited Tm^{3+} ions from the $^1I_6$ energy level to the ground state is responsible for the characteristic emission bands in the UV (340-360 nm), blue (450–416 nm), and red (635–680 nm) wavelength regions corresponding to the $^1I_6 \rightarrow {}^3F_4$, $^1D_2 \rightarrow {}^3H_6$, $^1D_2 \rightarrow {}^3F_4$, $^1G_4 \rightarrow {}^3H_6$, $^1G_4 \rightarrow {}^3F_4$ and $^3H_4 \rightarrow {}^3H_6$ transitions.

4. SAXS Measurements

4.1. Size Distribution

Raw data processing. Prior to the fitting of the SAXS data, the scattering images of both detectors were reduced and merged to a common scattering curve using the Python package pyFAI with a multi-geometry application. Here, q is the momentum transfer of the photon. Merging both images provides an overall increased q-range of the scattering curves. If the distance between the sample and the detector L_{SD} is determined accurately and the photon energy E_{ph} is known within the uncertainties given by the monochromator properties, the distance scale of the scattered reflex to the beam center on the detector plane in terms of pixel with size p can be converted into a q -scale via

$$q = \frac{4\pi E_{ph}}{hc} \sin\left(\frac{1}{2} \arctan \frac{n \cdot p}{L_{SD}}\right),$$ (eq. S1)

where h is Planck's constant, c is the speed of light and $n \cdot p$ is the pixel distance.

Results. The scattering curves obtained for **UCNP-BP1** and **UCNP-BP2** were fitted with a bipyramidal particle model where the edge length with a number-weighted lognormal size distribution and the height of the bipyramid, i.e., the peak-to-peak distance, are independent. The Python package CDEF[5] was used to model the particle shape as a cloud of 30000 discrete point virtual scatterers with equal scattering potential and to calculate the corresponding scattering curve using the Debye equation. The measured SAXS data were fitted by maximizing a log-likelihood function (objective function) assuming a Gaussian distribution of the measurement uncertainties. The uncertainties could be expanded by an adjustment parameter f to account for unknown uncertainty contributions. To maximize the objective function, first, a differential evolution algorithm was used to provide the starting point for the subsequent MCMC evaluation. An additional fit parameter was employed as a factor for the multiplication of the intensity of the background signal, thereby shifting it relative to the scattering curve of the sample. This allows for variable background correction. Thereby, 200 walkers with 10000 steps were used to sample the posterior distribution of the fitting parameters. The uncertainties of each parameter were assumed to be the total width of the respective distribution, excluding possible outliers, i.e., the quantiles 1 and 99.

UCNP-BP1. Within the uncertainties, the particle model provides a plausible description of the measured SAXS data. However, in the Guinier region, there are larger deviations observed between the measured data and the fit. This could possibly indicate a slight aggregation of the particles. The height $h = (43 \pm 2)\ \mathrm{nm}$ (see **Table 1** in the manuscript) supports the maximum Feret of $(36.5 \pm 2.5)\ \mathrm{nm}$ measured with TEM since the bipyramid lies on one of its sides, and the projection must therefore be less than the actual length/height (Fig. S5). The edge length of $R = (23.6 \pm 0.5)\ \mathrm{nm}$ (see **Table 1** in the manuscript) also supports the minimum Feret of $(23.5 \pm 1.3)\ \mathrm{nm}$ obtained by TEM.

UCNP-BP2. The maximum Feret of $(26.2 \pm 1.9)\ \mathrm{nm}$ measured with TEM agrees with the height of the bipyramide measured with SAXS (see **Table 1** in the manuscript).

4.2. Particle Number Concentration

Experimental details. For the determination of the particle concentration C, the samples were measured in rectangular capillaries (Hilgenberg GmbH, Germany) with a length of $80\ \mathrm{mm}$, a thickness of about $0.8\ \mathrm{mm}$, and a wall thickness of $125\ \mu\mathrm{m}$, which have a particularly homogeneous thickness along their longitudinal axis. During the SAXS measurements, the respective capillaries were scanned along the longitudinal axis, with a 20-second scattering image being measured every $0.5\ \mathrm{mm}$. Over a scan distance of about $20\ \mathrm{mm}$, around 40 scattering images were thus recorded per capillary. The advantage of this scanning measurement is that a sedimentation of the particles can be detected by a transmission gradient along the capillary axis and used for the subsequent uncertainty estimation. All samples show concentration variations of less than 2% along a capillary length of $10\ \mathrm{nm}$, so that sedimentation can be excluded. The pure suspension, i.e. the cyclohexane, was measured in a separate capillary. To determine the capillary thickness, Fluorinert FC-3283 with an optical absorption coefficient of $\frac{(2.44\pm 0.02)}{\mathrm{mm}}$ which is inert and does not mix with the sample, was filled into the bottom of each capillary, followed by the sample, and then the transmission through the capillary was measured.

The pre-factor N **(Tab. 1 – SI)** of the scattering curve is defined as

$$I(q) = N \cdot \int dr\, L(R,\sigma,r) \cdot V(r)^2 \cdot |F(q,r)|^2 = N \cdot model\ curve \qquad \text{(eq. S2)}$$

Here, $I(q)$ is the scattered intensity of the polydisperse particle ensemble with log-normal size distribution $L(r)$. The form factor $F(q,r)$ is calculated with CDEF.[5] $F(q,r)$ describes the particle shape which also depends on the specific particle dimension r. V is the particle volume. N is proportional to the number concentration (eq. S2) [8]. To experimentally determine N, the SAXS curves of the measured points were averaged for each capillary. The averaged SAXS curve was fitted with the scattering curve of the best suited particle model of the MCMC evaluation of the size determination to obtain N as a proportionality factor between the two curves. This result was subsequently used for the solution of the quadratic equation (Eq. 5). An MCMC algorithm provided uncertainties for the pre-factor N. Since in both examples there is some deviation between the best-fit model curve and the measured data in the Guinier region of the scattering curve, an additional independent uncertainty amount was added to the total uncertainty, which was assumed to be the deviation from the N value of the best-fit model curve and a separately performed Guinier fit.

The number concentration C was determined by using the particle's volume V determined from the MCMC evaluation of the size determination. Based on ICP-OES measurements, a molar mass M and an effective electron number Z of the particles at $8\ \mathrm{keV}$ were calculated.

Table S3. SAXS results of the particle number concentration C and mass density ρ_m of the bipyramidal particles including pre-factor N.

Sample	N / $10^{-13}\ \mathrm{nm}^{-7}$	c_m / $10^{-24}\ \frac{\mathrm{g}}{\mathrm{cm}^3}$	V / $10^3\ \mathrm{nm}^3$	Z / 1	M / $\frac{\mathrm{g}}{\mathrm{mol}}$	$c/$ $10^{14}\ \mathrm{cm}^{-3}$	ρ_m / $\frac{\mathrm{g}}{\mathrm{cm}^3}$
UCNP-BP1	5.8 ± 1.3	3.935	8.0 ± 0.2	75.5 ± 0.3	172.08 ± 0.03	1.3 ± 0.2	3.9 ± 0.5
UCNP-BP2	17 ± 4	3.88	3.52 ± 0.09	75.2 ± 0.2	172.10 ± 0.03	2.4 ± 0.4	4.7 ± 0.7

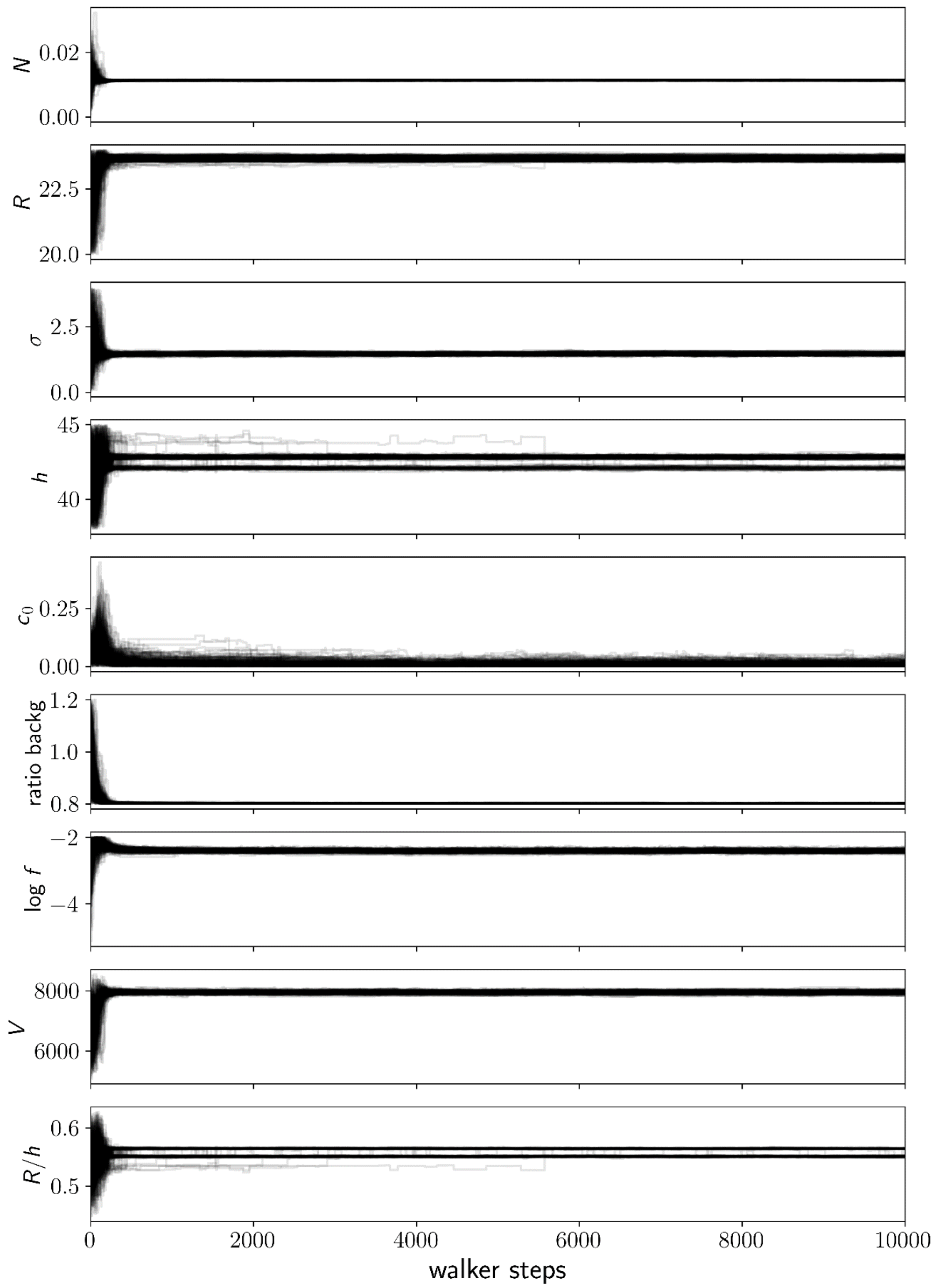

Fig. S7: Chain plot for each fit parameter of the MCMC evaluation of the size distribution of UCNP-BP1, showing the values of all walkers taking 10000 steps used to maximize the objective function. Since the walkers begin their exploration of the parameter space distributed around the supposed maximum of the posterior, they require a certain burn-in phase until they begin to continuously move around the maximum. After the burn-in phase, the positions of the walker are no longer auto-correlated with its starting position, and thus provide independent results. To correct the burn-in phase for UCNP-BP1, the first 3000 steps were discarded, although the specific boundary is different for each individual parameter on closer analysis of the chain plot. The fit parameter ratio backg describes the coefficient that is multiplied by the background curve for its optimum subtraction from the sample curve. c_0 is an additional constant scattering background and $\log f$ is a parameter to

extend the experimental uncertainty. Interestingly, the particle volume V remains constant although the particle height h is bimodal. This could also indicate certain symmetries with respect to the area and shape of the irradiated cross-section of the isoptropically oriented particles.

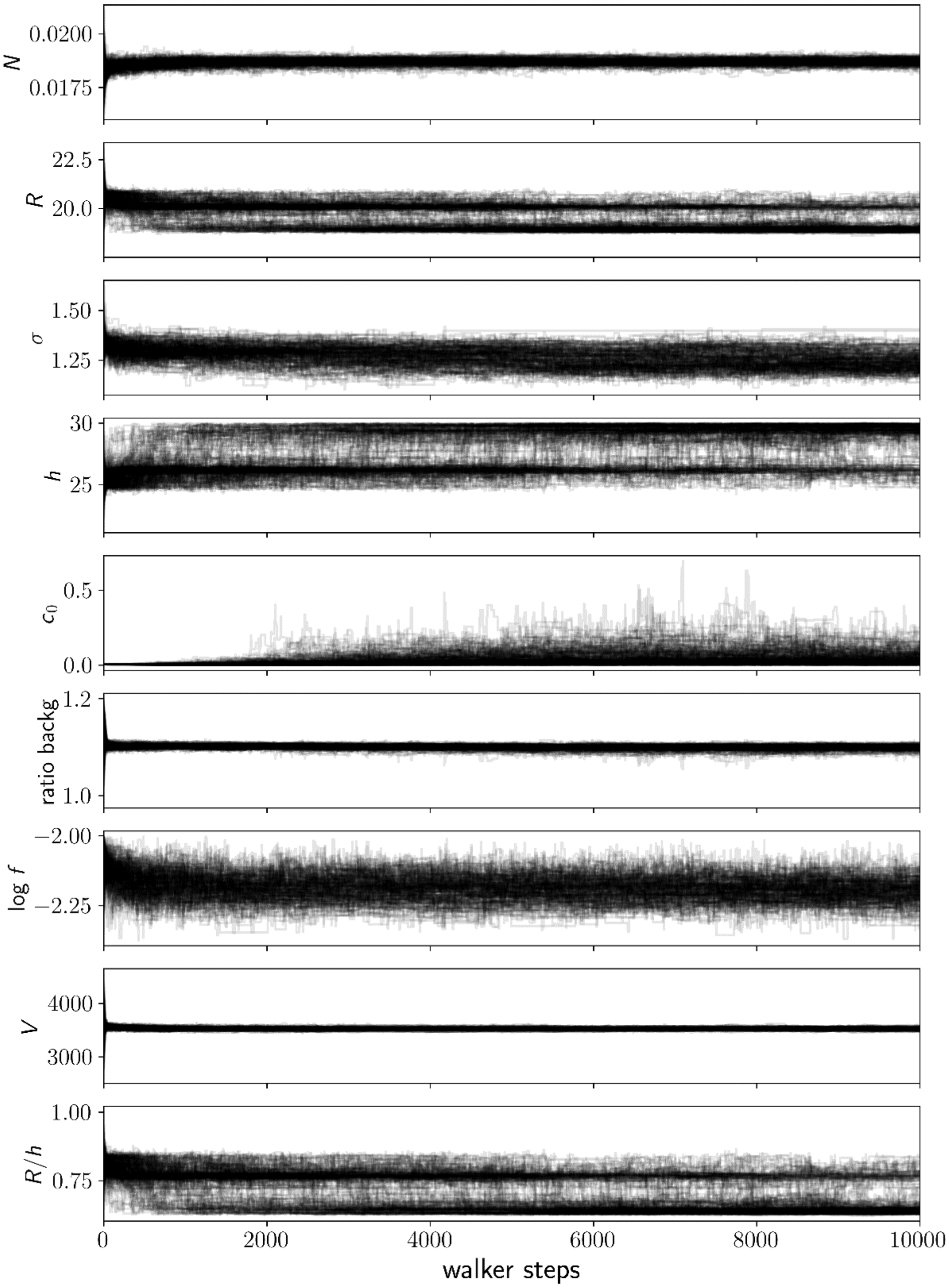

Fig. S8: Chain plot for each fit parameter of the MCMC evaluation of UCNP-BP2, showing the values of all walkers taking 10000 steps and trying to maximize the objective function. The burn-in phase requires approx. 3000 steps for UCNP-BP2. For the variables R, h the diagram again shows multimodalities in the particle distribution. It is known from the TEM data that only parameter sets with an edge length $R < 20$ nm are meaningful. Therefore, all other parameter sets are discarded for the analysis of the size distribution and particle number concentration.

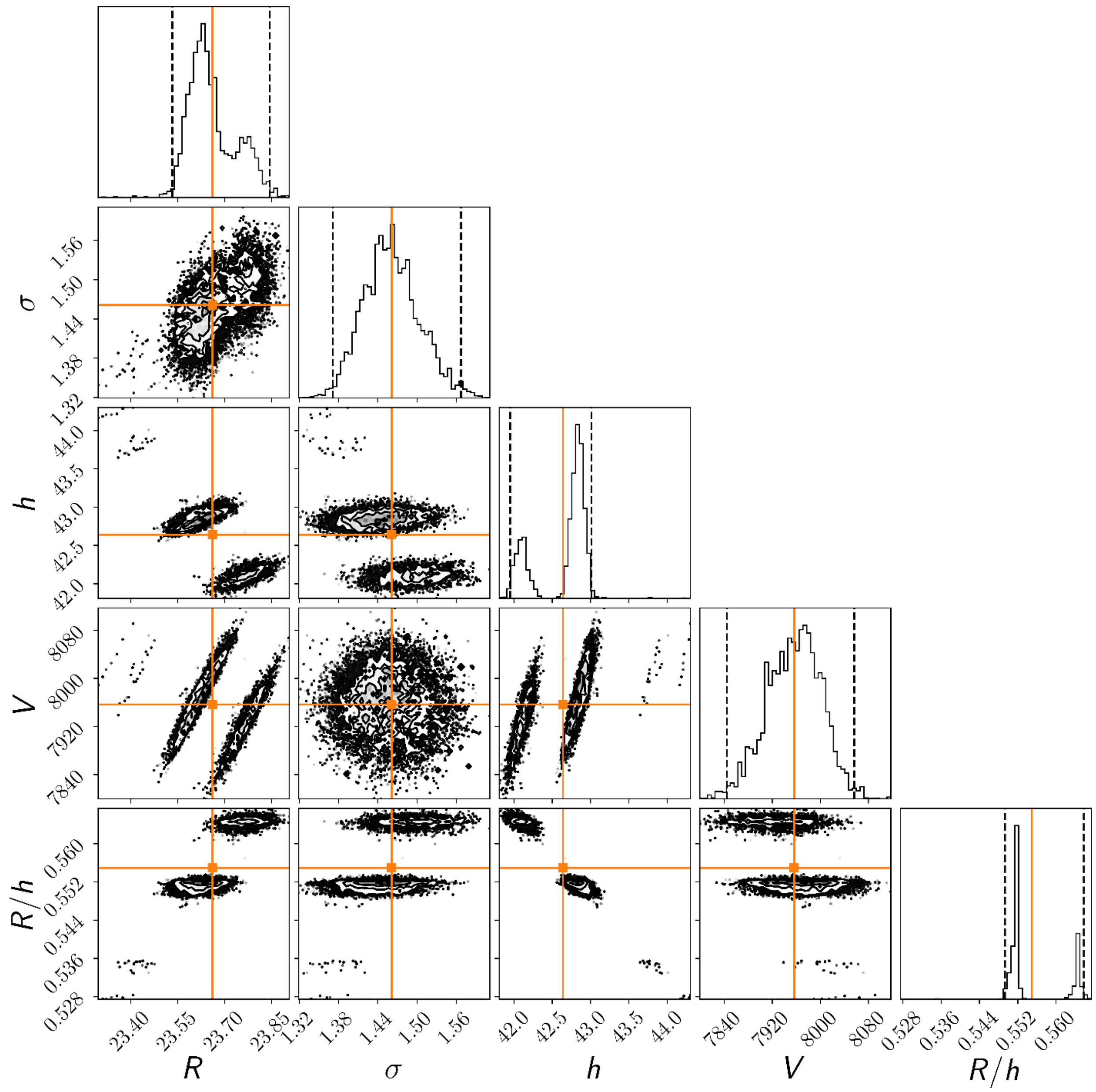

Fig. S9: Corner plot of the MCMC evaluation of the size distribution of UCNP-BP1 after the burn-in phase (discarding the first 3000 steps). The probability distributions of the individual parameters are shown, with the mean value being visualized as an orange line and the quantiles 1 and 99 as dashed black lines serving as uncertainties. All covariances between the parameters are also shown. Particle volume V as well as the aspect ratio $\frac{R}{h}$ are not actual fit parameters but were calculated to show additional covariances.

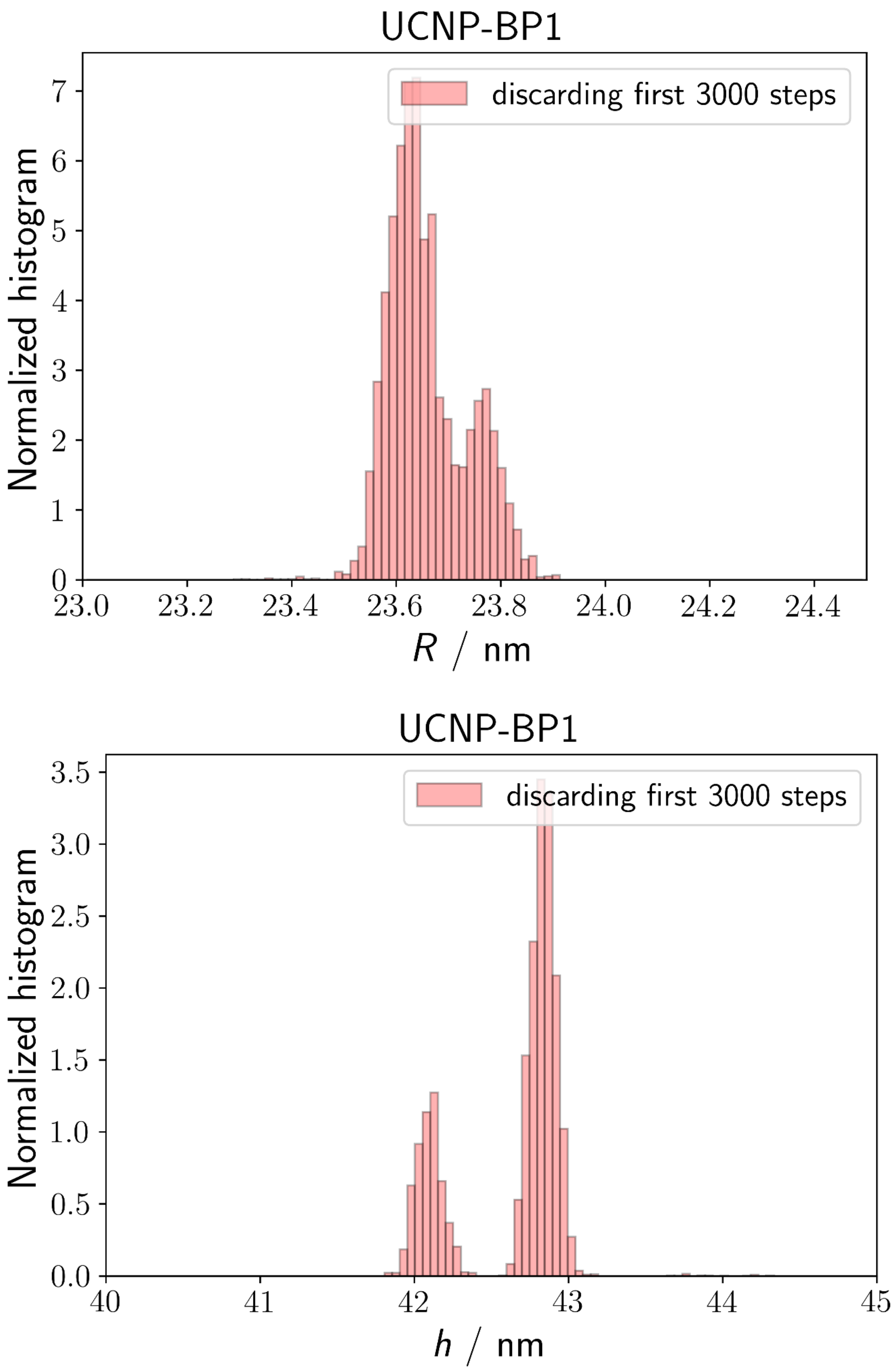

Fig. S10: Rebinned histograms of the edge length R and the height h of the MCMC evaluation of the size distribution of UCNP-BP1 after the burn-in phase (neglecting the first 3000 steps). The height h is bimodally distributed. The percentiles 1 to 99 serve as uncertainty. The respective mean values are used as the measurement result.

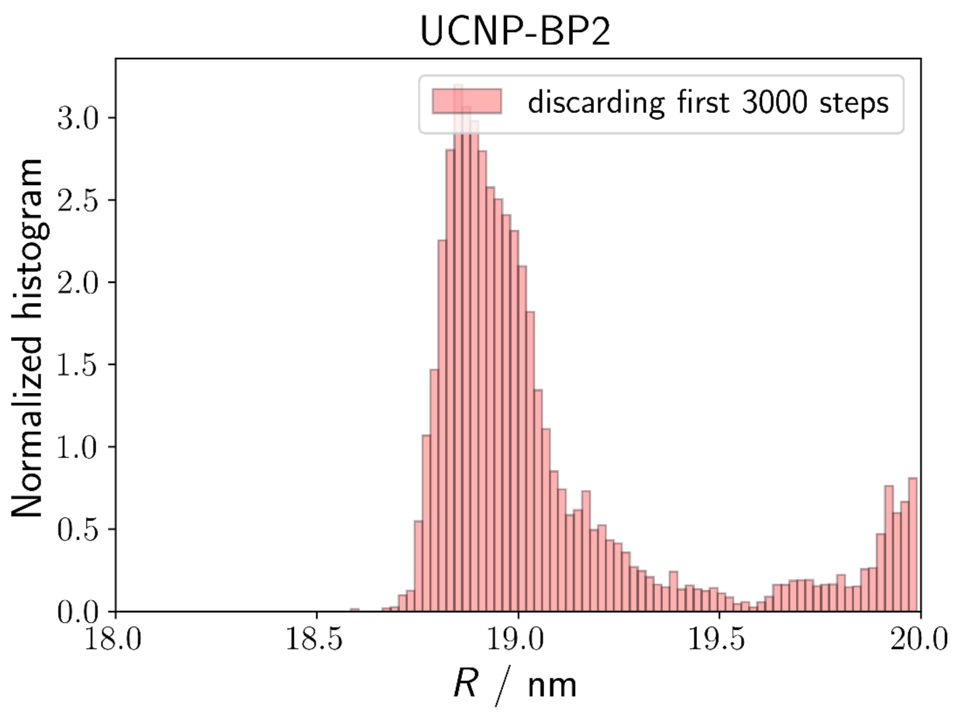

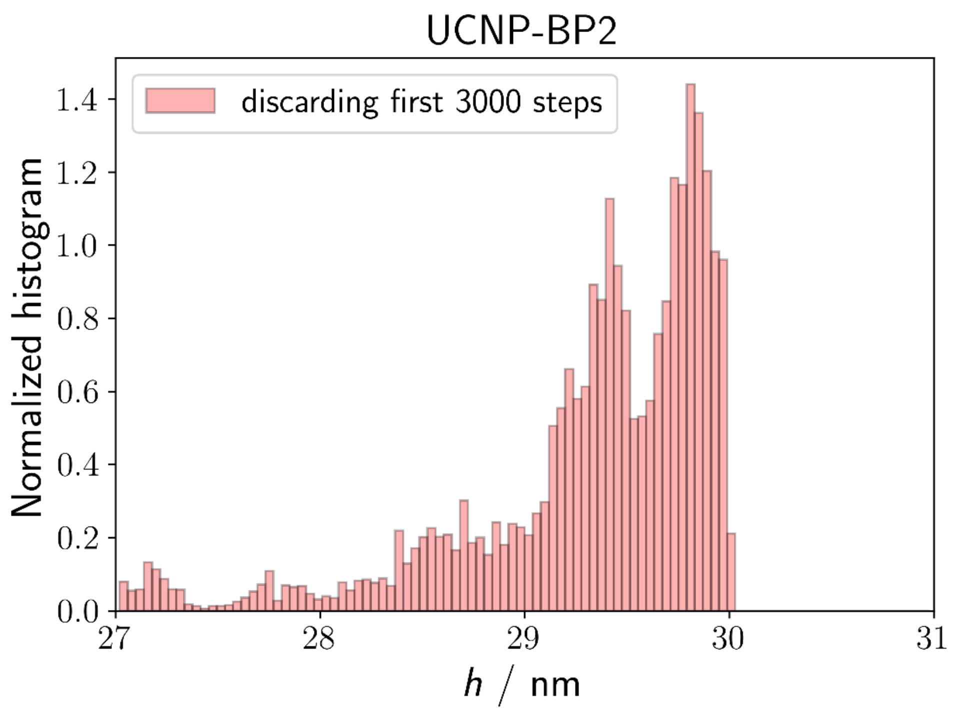

Fig. S11: Rebinned histograms of the edge length R and the height h of the MCMC evaluation of the size distribution of UCNP-BP2 after the burn-in phase (neglecting the first 3000 steps). The percentiles 1 to 99 serve as uncertainty. The respective mean values are used as the measurement result.

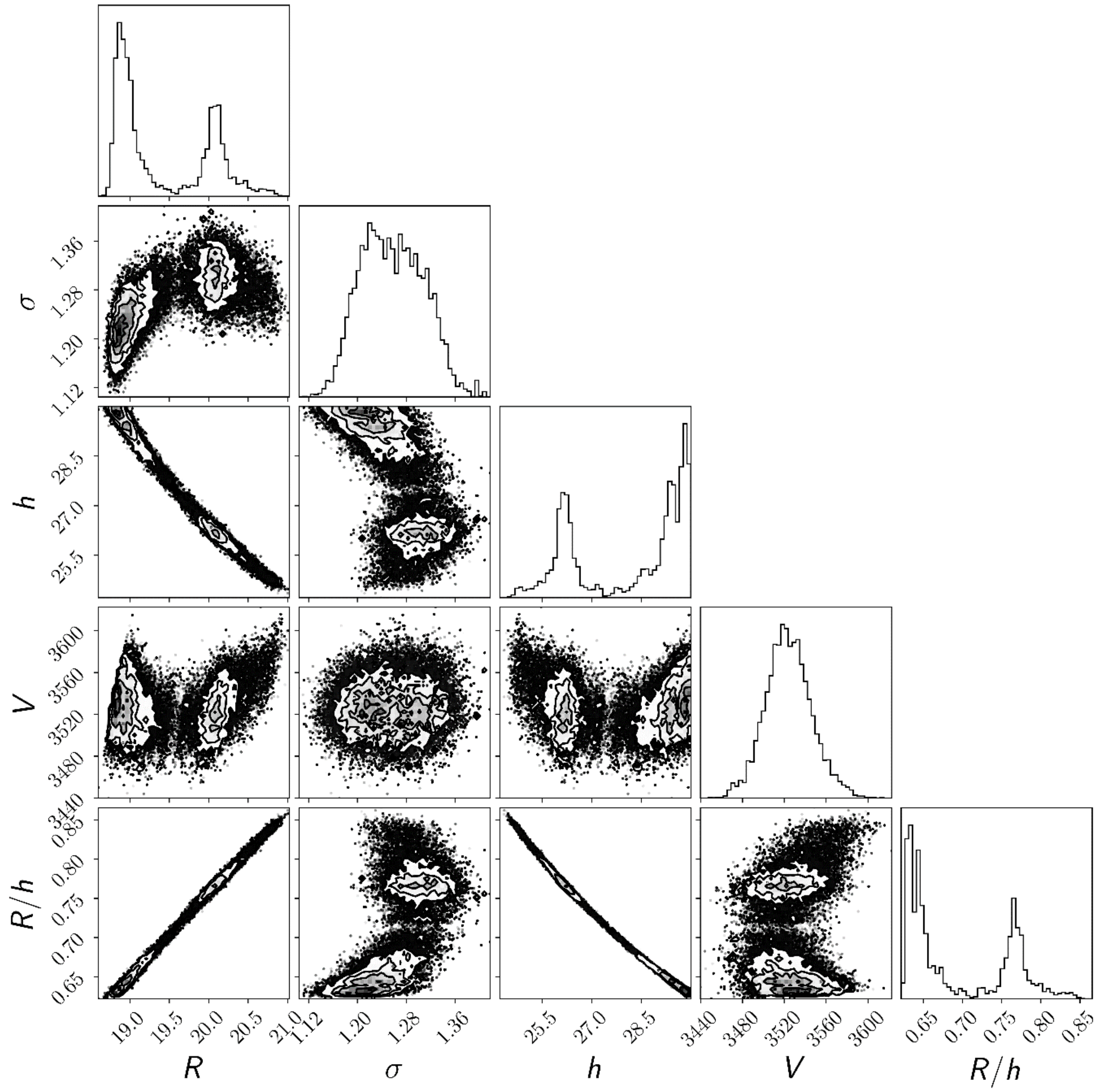

Fig. S12: Corner plot of the MCMC evaluation of UCNP-BP2 after the burn-in phase (discarding the first 3000 steps). All covariances between the parameters are also shown. Particle volume V*, again, was not an actual fit parameter, but was calculated from the variables* R*,* σ*,* h *to show covariances.*

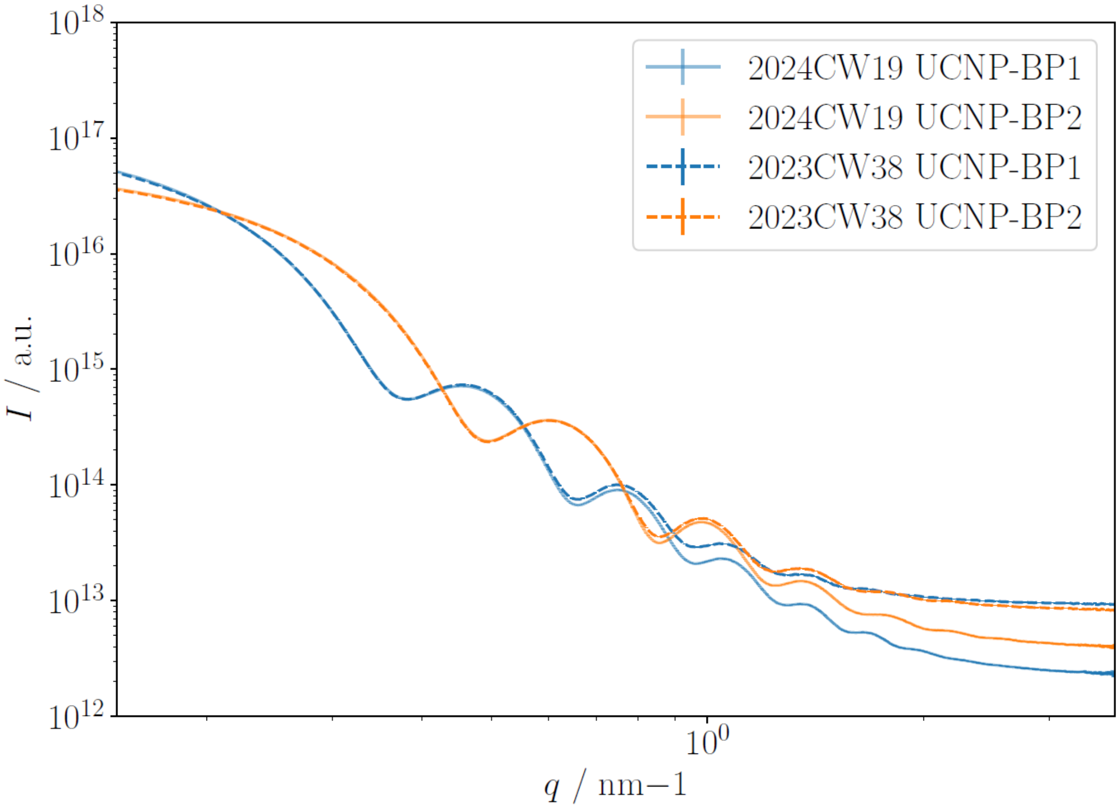

Fig. S13: *SAXS scattering curves showing scattered intensity I as a function of momentum transfer q for UCNP-BP1 (blue) and UCNP-BP2 (orange). Two measurements (solid line and dashed line) were carried out at intervals of 8 months. The differences in the high q-region most likely originate from the subtraction of the background.*

Further details of the calculation of the particle number concentration C using the mass concentration c_m:

$$c_m = c \cdot \rho_m \cdot V, \quad \text{(eq. S3)}$$

$$c = N/(r_e^2 \cdot \Delta\rho_e^2), \quad \text{(eq. S4)}$$

$$\Delta\rho_e = \frac{\rho_m}{M} \cdot Z \cdot N_A - \rho_c. \quad \text{(eq. S5)}$$

Here, C is the number concentration, c_m the mass concentration experimentally corrected by TGA measurements, ρ_m is the mass density, V is the particle volume, and N is a pre-factor of the scattering curve. r_e equals the Thomson scattering length, $\Delta\rho_e$ is the electron density contrast, M is the molar mass, Z is the averaged electron charge, N_A is the Avogadro constant, and ρ_c is the electron density of the solvent, here cyclohexane.

Equations (S3) – (S5) lead to a quadratic equation for the particle number concentration C:

$$C^2 + p \cdot C + q = 0, \quad \text{(eq. S6)}$$

$$\text{with } p = \frac{(-2 \cdot c_m \cdot N_A \cdot Z \cdot r_e^2 \cdot \rho_c - N \cdot V \cdot M)}{r_e^2 \cdot \rho_c^2 \cdot V \cdot M}, \quad \text{(eq. S7)}$$

$$\text{and } q = \frac{(c_m \cdot N_A \cdot Z)^2}{(\rho_c \cdot V \cdot M)^2}. \quad \text{(eq. S8)}$$

5. References:

[1] Krumrey, Michael. "Design of a four-crystal monochromator beamline for radiometry at BESSY II." Journal of synchrotron radiation 5.1 (1998): 6-9.

[2] Wernecke, Jan, et al. "Characterization of an in-vacuum PILATUS 1M detector." Journal of Synchrotron Radiation 21.3 (2014): 529-536.

[3] Skroblin, D., et al. "Vacuum-compatible photon-counting hybrid pixel detector for wide-angle x-ray scattering, x-ray diffraction, and x-ray reflectometry in the tender x-ray range." *Review of Scientific Instruments* 91.2 (2020).

[4] Kieffer, Jérôme, and Dimitrios Karkoulis. "PyFAI, a versatile library for azimuthal regrouping." Journal of Physics: Conference Series. Vol. 425. No. 20. IOP Publishing, 2013.

[5] Deumer, Jérôme, et al. "Small-angle X-ray scattering: characterization of cubic Au nanoparticles using Debye's scattering formula." *Journal of Applied Crystallography* 55.4 (2022): 993-1001.

[6] Foreman-Mackey, Daniel, et al. "emcee: the MCMC hammer." *Publications of the Astronomical Society of the Pacific* 125.925 (2013): 306.

[7] Minelli, Caterina, et al. "Versailles project on advanced materials and standards (VAMAS) interlaboratory study on measuring the number concentration of colloidal gold nanoparticles." Nanoscale 14.12 (2022): 4690-4704.

[8] Schavkan, Alexander, et al. "Number concentration of gold nanoparticles in suspension: SAXS and spICPMS as traceable methods compared to laboratory methods." Nanomaterials 9.4 (2019): 502.
